# Supplementary material for: Design, Synthesis and Evaluation of Novel Trichloromethyl Dichlorophenyl Triazole Derivatives as Potential Safener
Source: Biomolecules. 2019 Sep 1;9(9):438. doi: 10.3390/biom9090438 (PMC6770657; doi:10.3390/biom9090438)
Supplement: Supplementary file 1 [file biomolecules-09-00438-s001.pdf]

SUPPORTING INFORMATION

Design, Synthesis and Evaluation of Novel Trichloromethyl Dichlorophenyl Triazole Derivatives as Potential Safener

Ke-Liang Guo †, Li-Xia Zhao †, Zi-Wei Wang, Shu-Zhe Rong, Xiao-Lin Zhou, Shuang Gao, Ying Fu * and Fei Ye *

Department of Applied Chemistry, College of Science, Northeast Agricultural University, Harbin, 150030, China;

* Correspondence: fuying@neau.edu.cn (Y. F.); yefei@neau.edu.cn (F. Y.)

† These authors contributed equally to this work.

Contents:

1. The molecular structure and spectra of representative compound **5a** 3
2. The molecular structure and spectra of representative compound **5b**............................5
3. The molecular structure and spectra of representative compound **5c** 7
4. The molecular structure and spectra of representative compound **5d**............................9
5. The molecular structure and spectra of representative compound **5e** 11
6. The molecular structure and spectra of representative compound **5f**...........................13
7. The molecular structure and spectra of representative compound **5g**..........................15
8. The molecular structure and spectra of representative compound **5h**..........................17
9. The molecular structure and spectra of representative compound **5i**...........................19
10. The molecular structure and spectra of representative compound **5j**...........................21
11. The molecular structure and spectra of representative compound **5k**..........................23
12. The molecular structure and spectra of representative compound **5l**...........................25
13. The molecular structure and spectra of representative compound **5m**.........................27
14. The molecular structure and spectra of representative compound **5n**..........................29
15. The molecular structure and spectra of representative compound **5o**..........................31
16. The molecular structure and spectra of representative compound **5p**..........................33
17. The molecular structure and spectra of representative compound **5q**..........................35
18. The molecular structure and spectra of representative compound **5r**...........................37
19. The molecular structure and spectra of representative compound **5s** 39
20. The molecular structure and spectra of representative compound **5t**...........................41
21. The molecular structure and spectra of representative compound **5u**..........................43

1. The molecular structure and spectra of representative compound **5a**

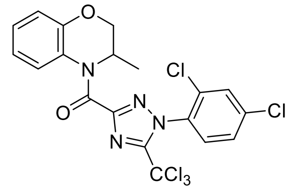

(1-(2,4-dichlorophenyl)-5-(trichloromethyl)-1*H*-1,2,4-triazol-3-yl)(3-methyl-2,3-dihydro-4*H*-benzo[*b*][1,4]oxazin-4-yl)methanone

$C_{19}H_{13}Cl_5N_4O_2$

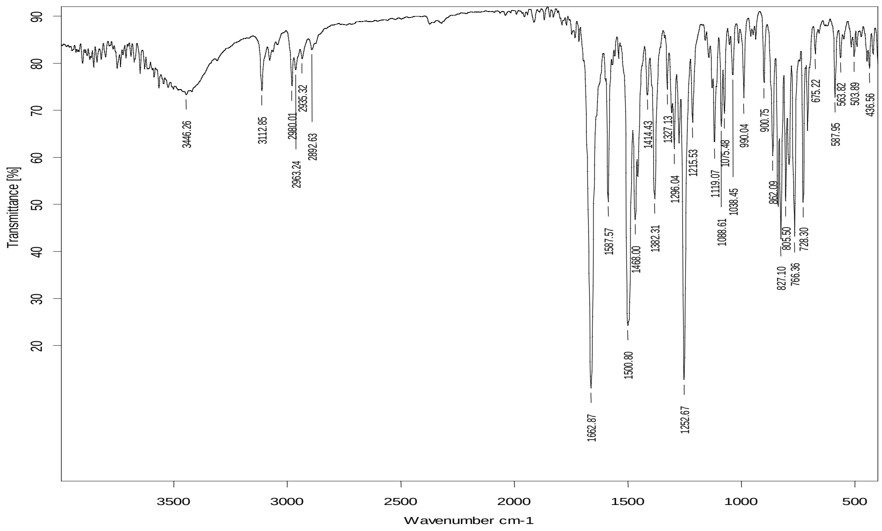

The IR spectrogram of compound **5a**

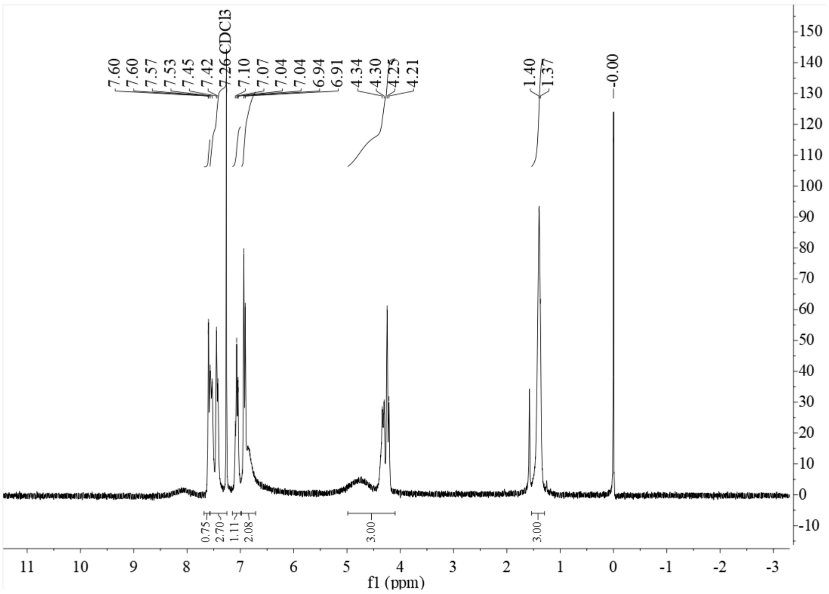

The ^{1}H NMR spectrogram of compound **5a**

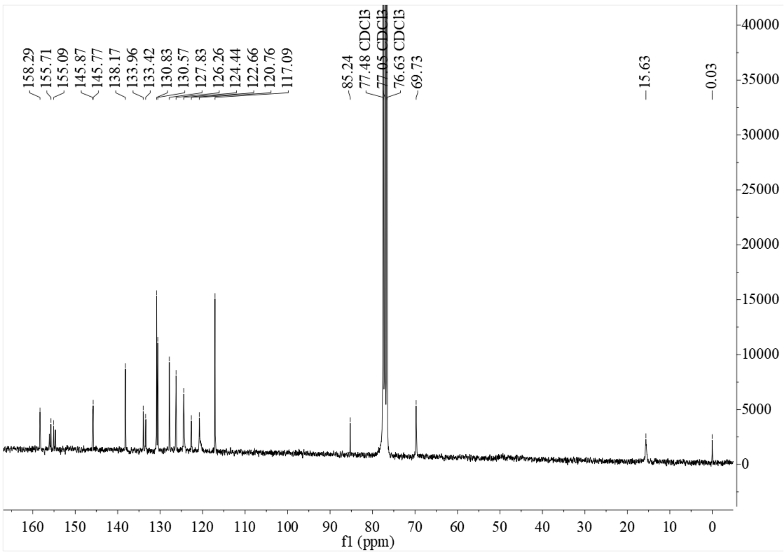

The ^{13}C NMR spectrogram of compound **5a**

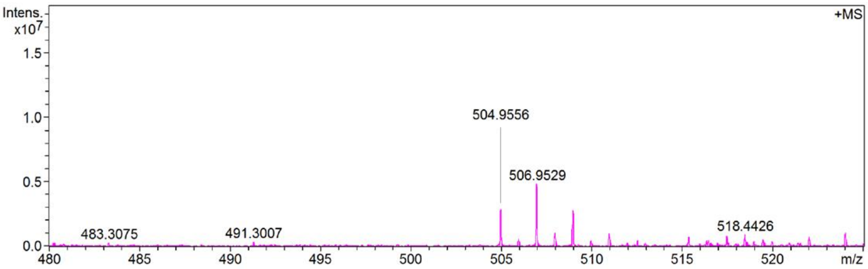

The HRMS spectrogram of compound **5a**

2. The molecular structure and spectra of representative compound **5b**

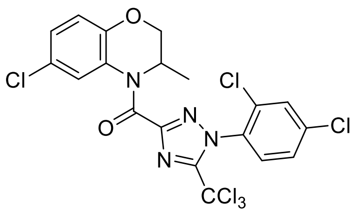

(6-chloro-3-methyl-2,3-dihydro-4*H*-benzo[*b*][1,4]oxazin-4-yl)(1-(2,4-dichlorophenyl)-5-(trichloromethyl)-1*H*-1,2,4-triazol-3-yl)methanone

$C_{19}H_{12}Cl_6N_4O_2$

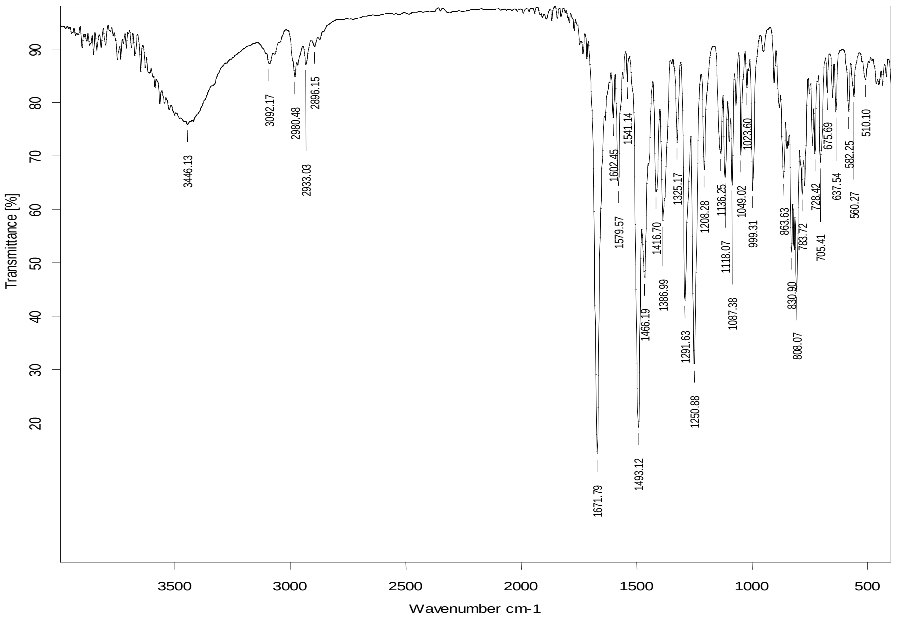

The IR spectrogram of compound **5b**

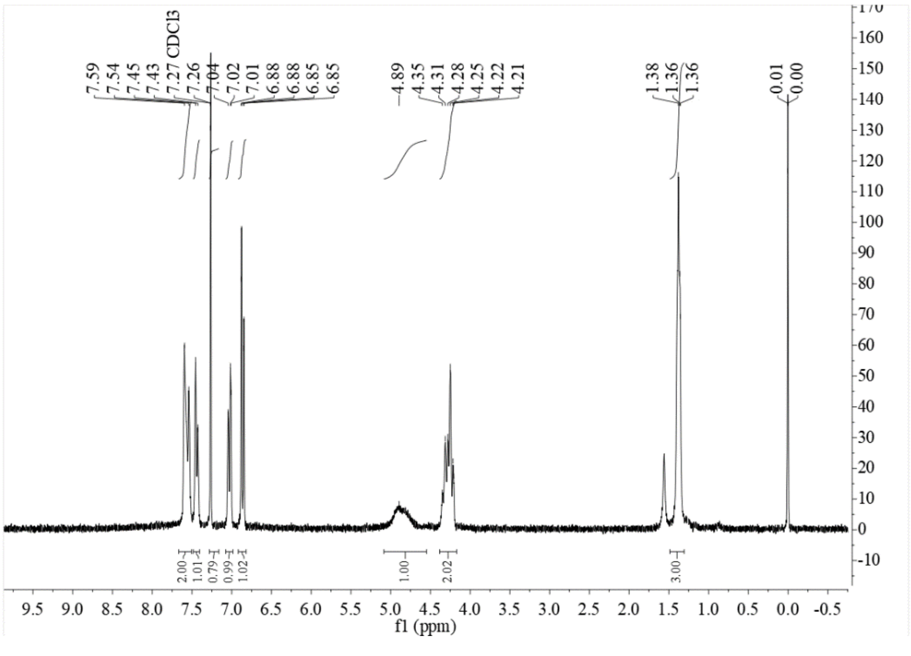

The ^{1}H NMR spectrogram of compound **5b**

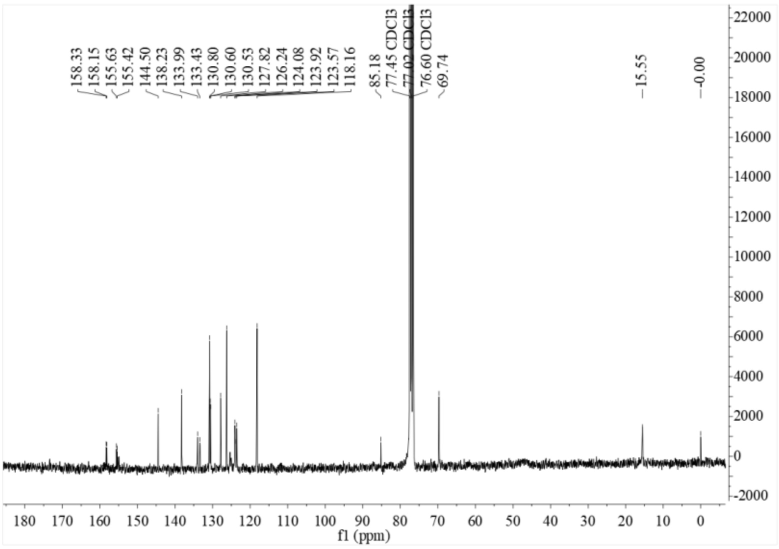

The ^{13}C NMR spectrogram of compound **5b**

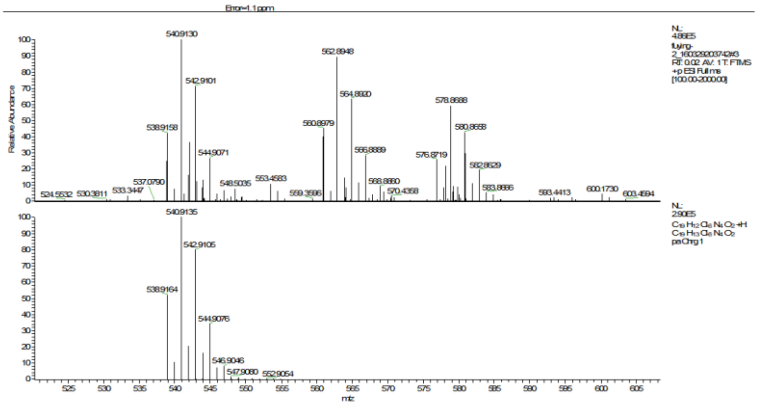

The HRMS spectrogram of compound **5b**

3. The molecular structure and spectra of representative compound **5c**

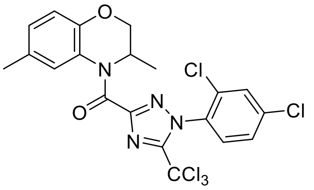

(1-(2,4-dichlorophenyl)-5-(trichloromethyl)-1*H*-1,2,4-triazol-3-yl)(3,6-dimethyl-2,3-dihydro-4*H*-benzo[*b*][1,4]oxazin-4-yl)methanone

$C_{20}H_{15}Cl_5N_4O_2$

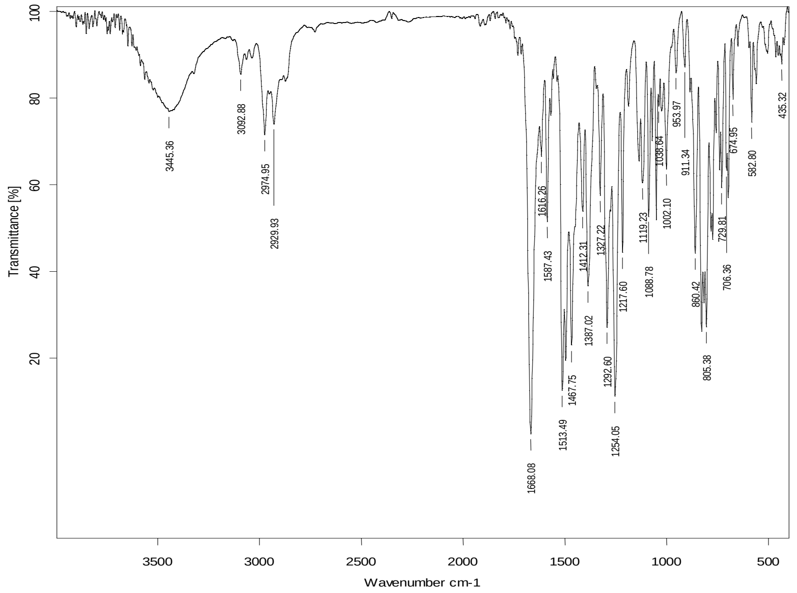

The IR spectrogram of compound **5c**

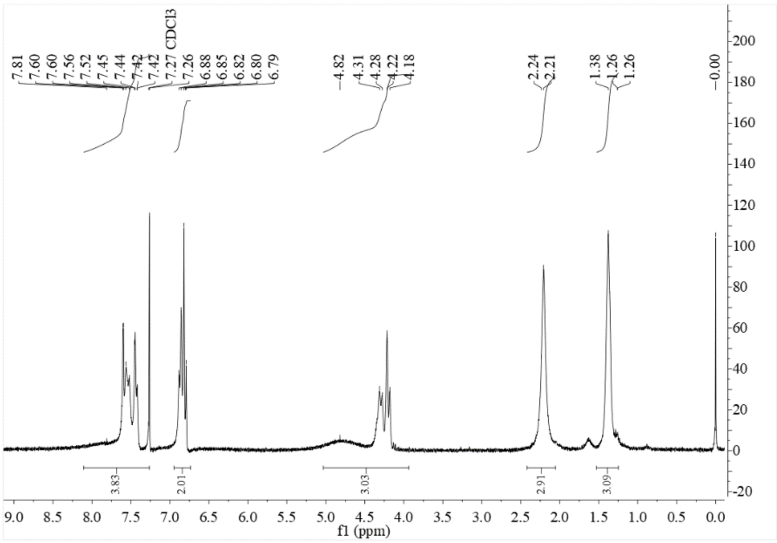

The ^{1}H NMR spectrogram of compound **5c**

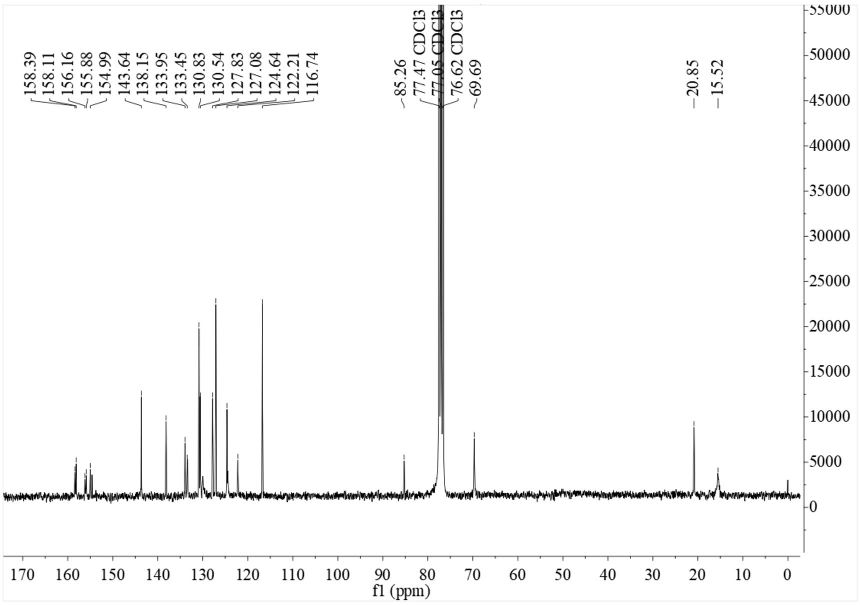

The ^{13}C NMR spectrogram of compound **5c**

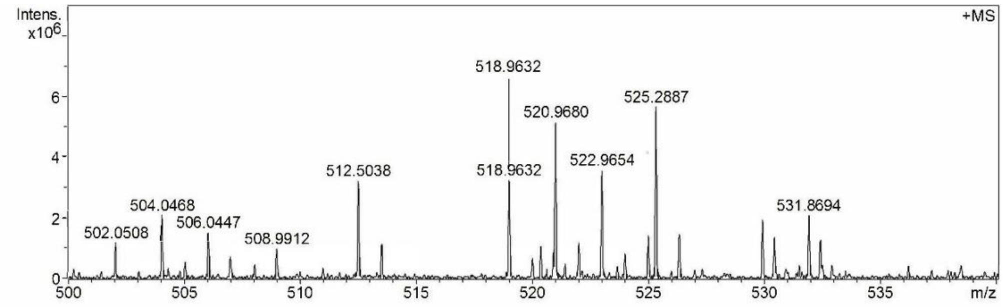

The HRMS spectrogram of compound **5c**

4. The molecular structure and spectra of representative compound **5d**

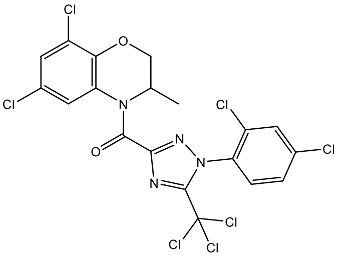

(6,8-dichloro-3-methyl-2,3-dihydro-4*H*-benzo[*b*][1,4]oxazin-4-yl)(1-(2,4-dichlorophenyl)-5-(trichloromethyl)-1*H*-1,2,4-triazol-3-yl)methanone

$C_{19}H_{11}Cl_7N_4O_2$

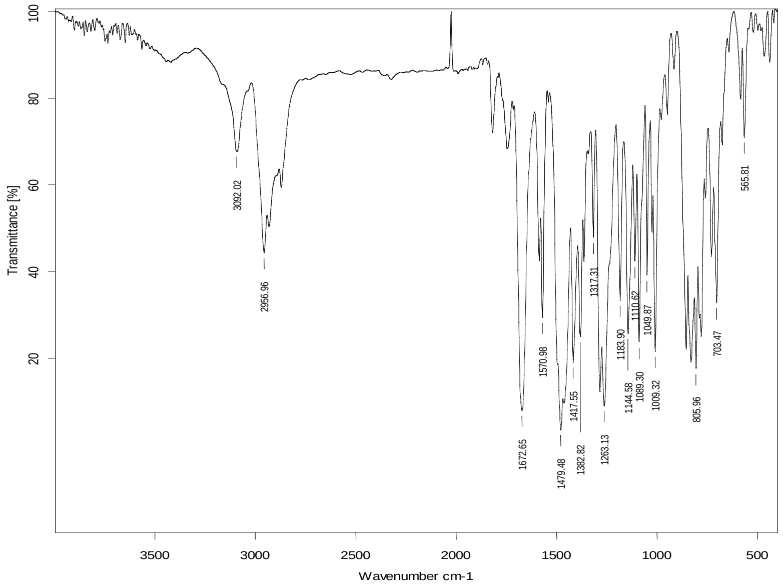

The IR spectrogram of compound **5d**

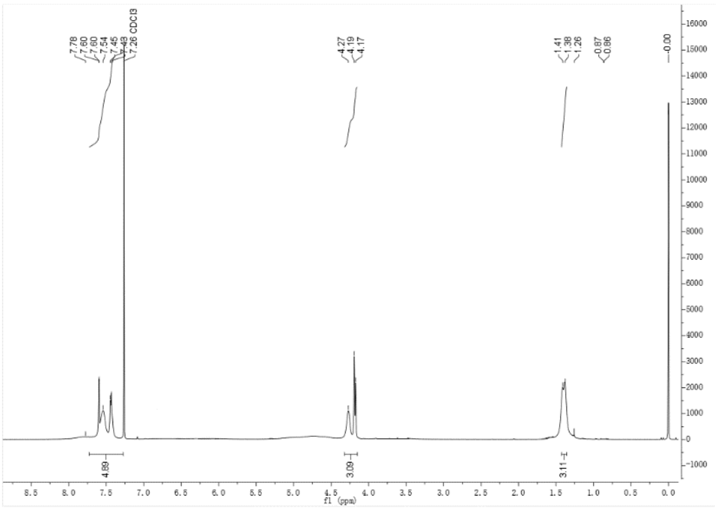

The ^{1}H NMR spectrogram of compound **5d**

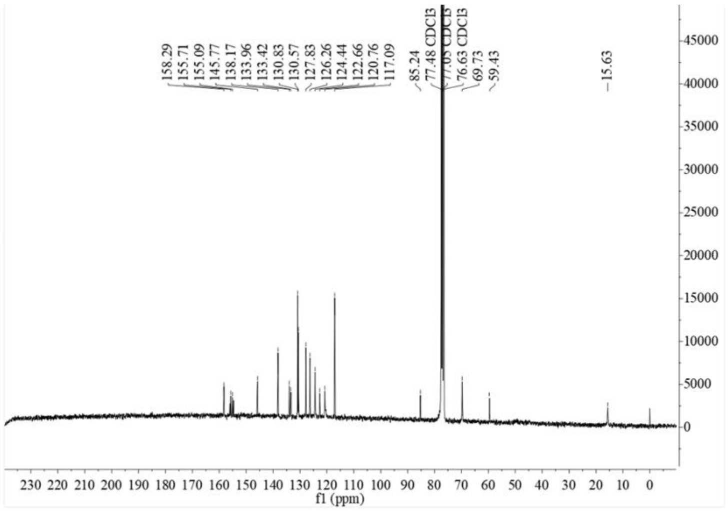

The ^{13}C NMR spectrogram of compound **5d**

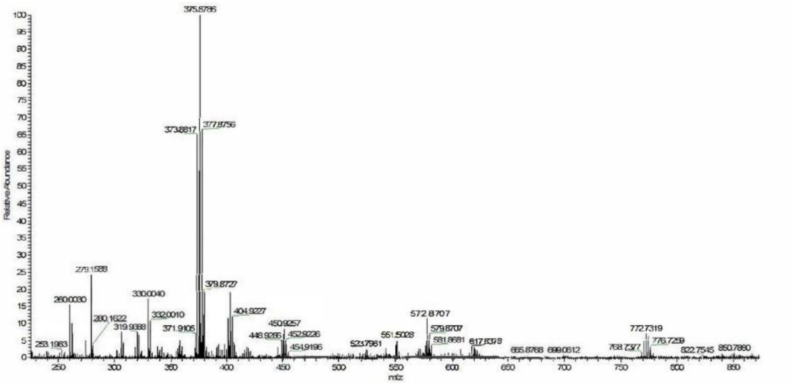

The HRMS spectrogram of compound **5d**

5. The molecular structure and spectra of representative compound **5e**

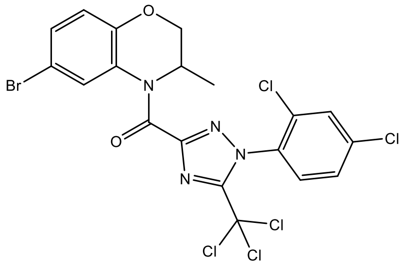

(6-bromo-3-methyl-2,3-dihydro-4*H*-benzo[*b*][1,4]oxazin-4-yl)(1-(2,4-dichlorophenyl)-5-(trichloromethyl)-1*H*-1,2,4-triazol-3-yl)methanone

$C_{19}H_{12}BrCl_5N_4O_2$

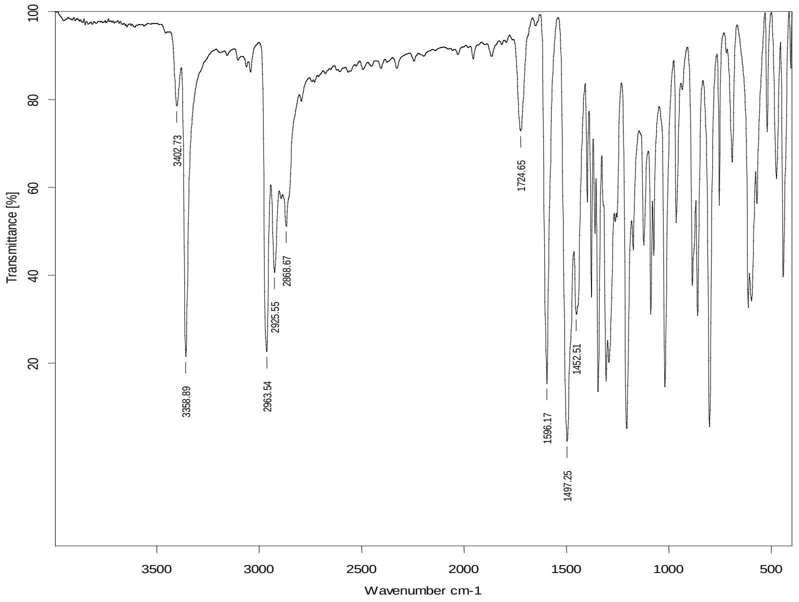

The IR spectrogram of compound **5e**

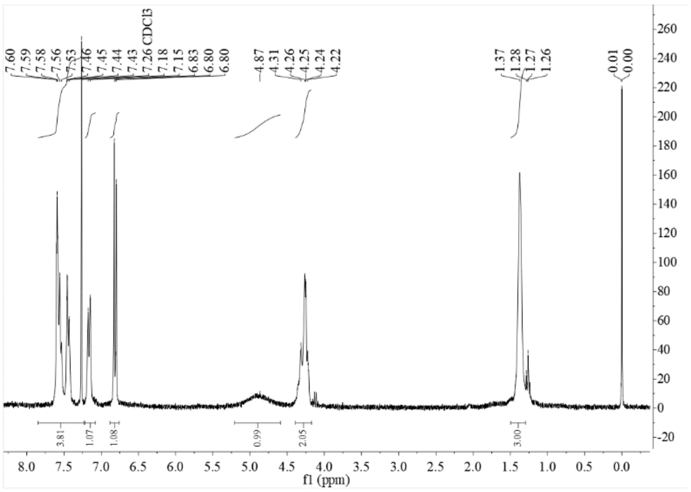

The ^{1}H NMR spectrogram of compound **5e**

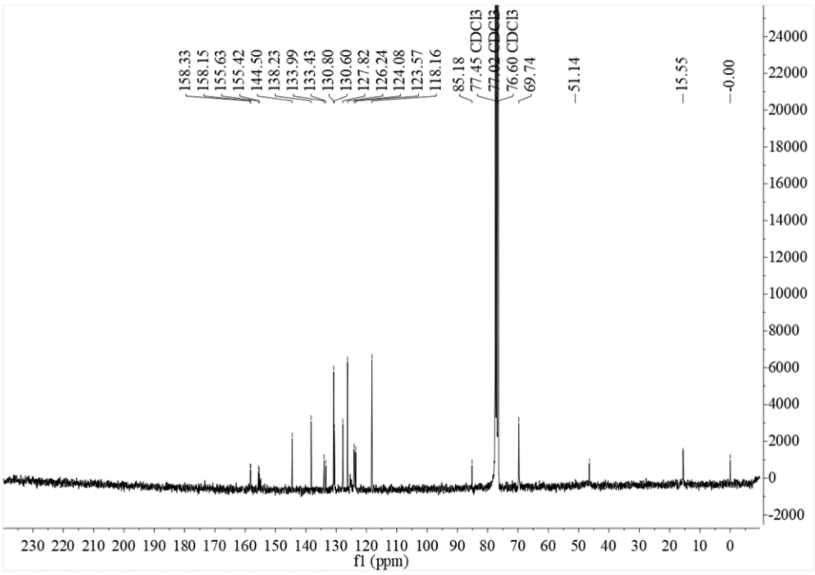

The ^{13}C NMR spectrogram of compound **5e**

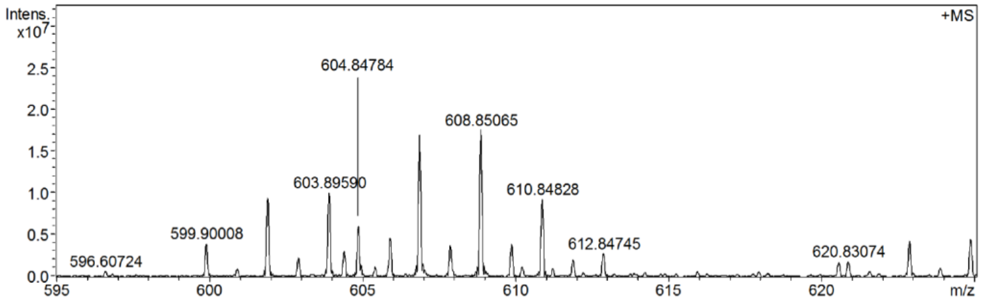

The HRMS spectrogram of compound **5e**

6. The molecular structure and spectra of representative compound **5f**

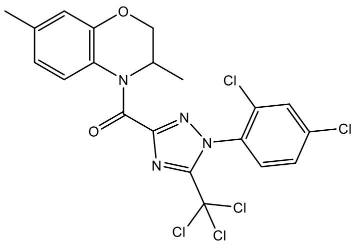

(1-(2,4-dichlorophenyl)-5-(trichloromethyl)-1*H*-1,2,4-triazol-3-yl)(3,7-dimethyl-2,3-dihydro-4*H*-benzo[*b*][1,4]oxazin-4-yl)methanone

$C_{20}H_{15}Cl_5N_4O_2$

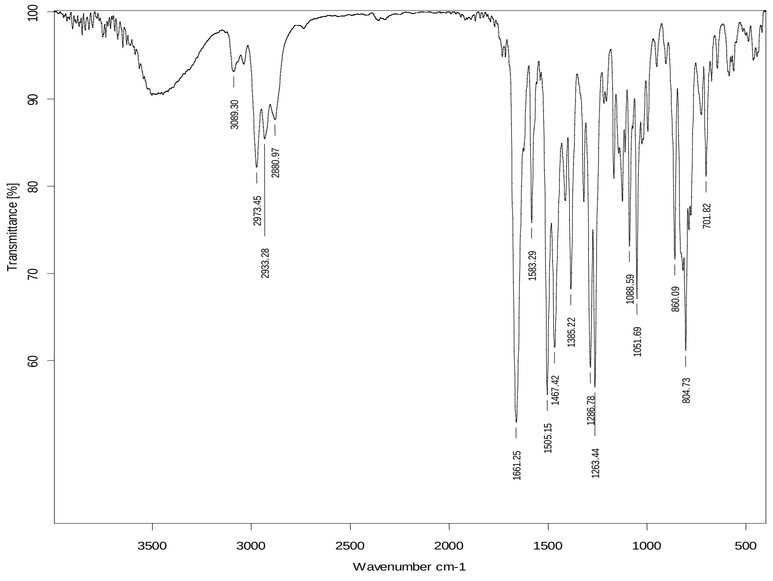

The IR spectrogram of compound **5f**

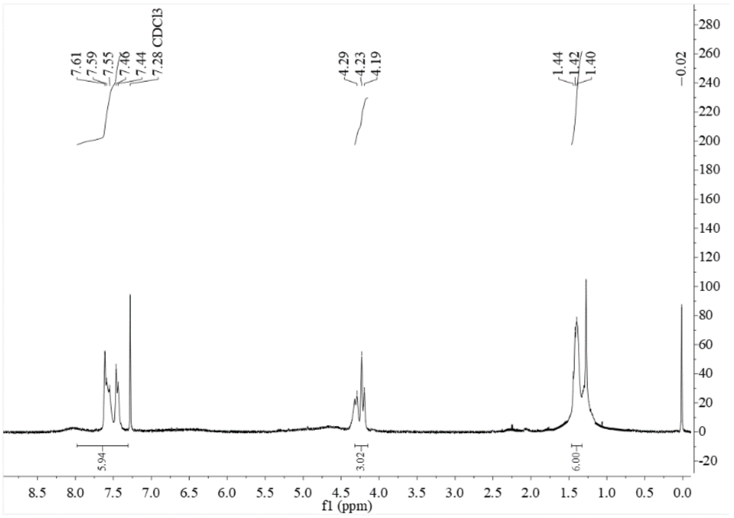

The ^{1}H NMR spectrogram of compound **5f**

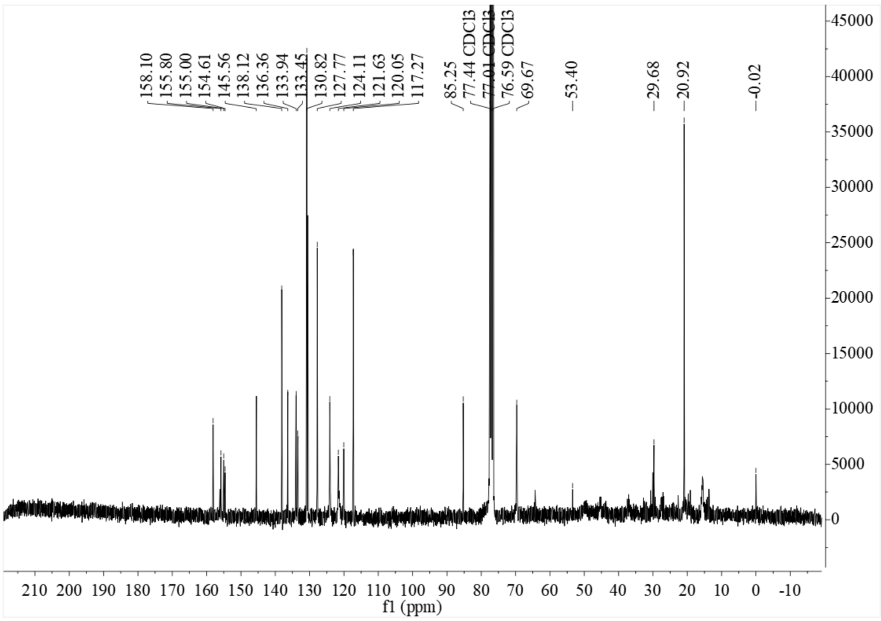

The ^{13}C NMR spectrogram of compound **5f**

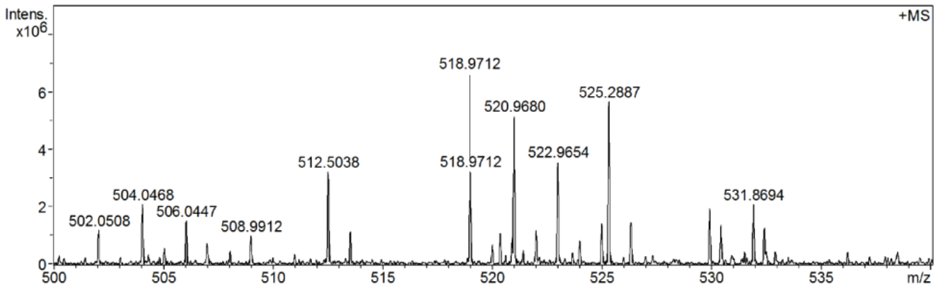

The HRMS spectrogram of compound **5f**

7. The molecular structure and spectra of representative compound **5g**

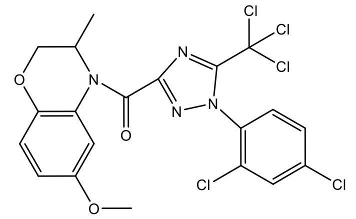

(1-(2,4-dichlorophenyl)-5-(trichloromethyl)-1*H*-1,2,4-triazol-3-yl)(6-methoxy-3-methyl-2,3-dihydro-4*H*-benzo[*b*][1,4]oxazin-4-yl)methanone

$C_{20}H_{15}Cl_5N_4O_3$

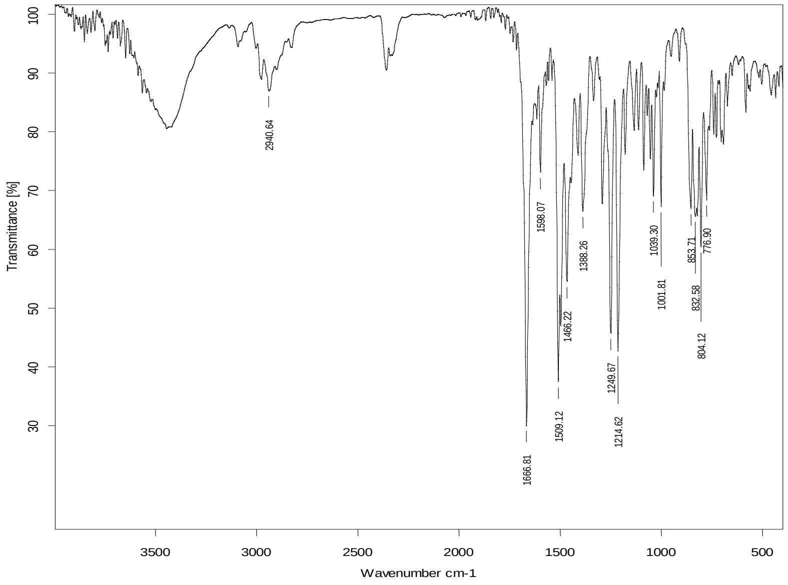

The IR spectrogram of compound **5g**

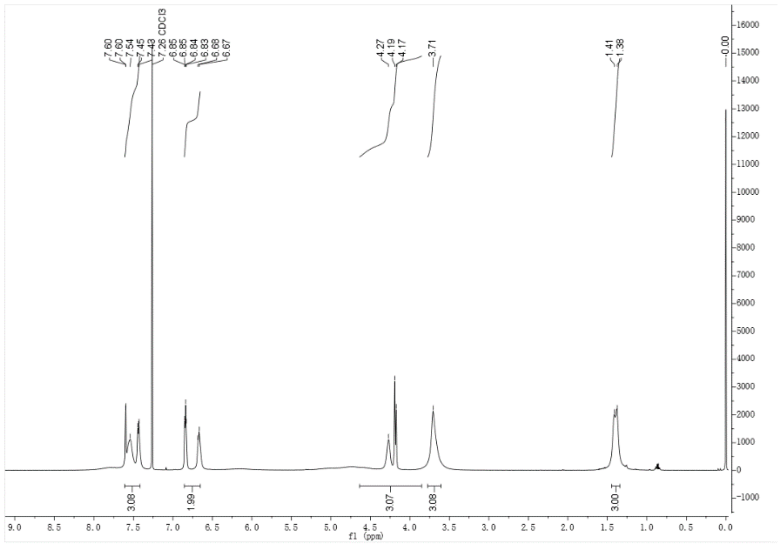

The ^{1}H NMR spectrogram of compound **5g**

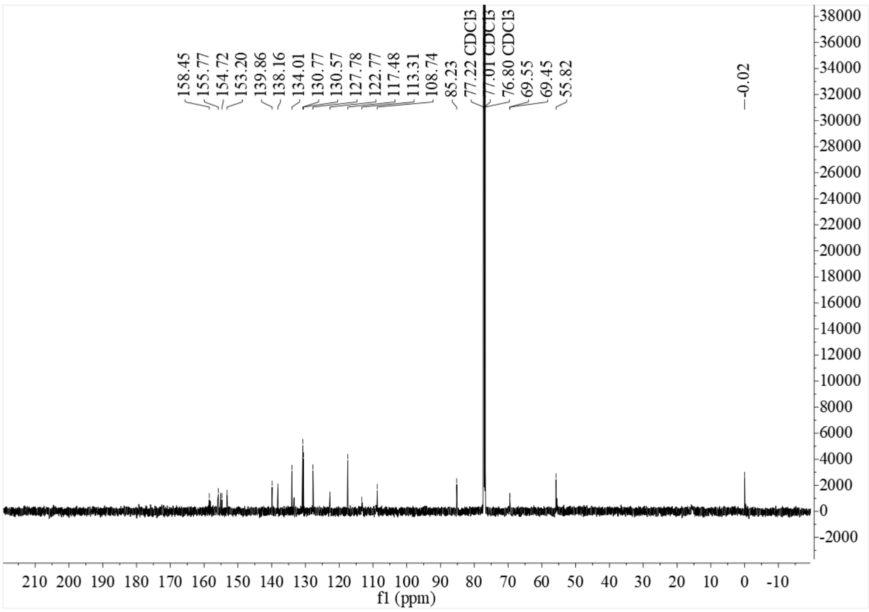

The ^{13}C NMR spectrogram of compound **5g**

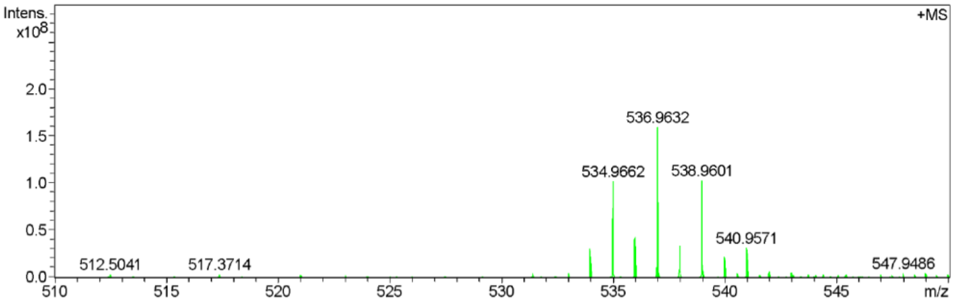

The HRMS spectrogram of compound **5g**

8. The molecular structure and spectra of representative compound **5h**

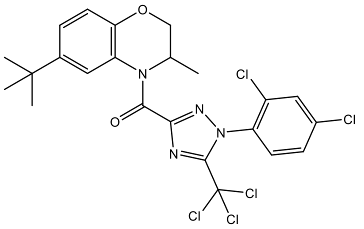

(6-(*tert*-butyl)-3-methyl-2,3-dihydro-4*H*-benzo[*b*][1,4]oxazin-4-yl)(1-(2,4-dichlorophenyl)-5-(trichloromethyl)-1*H*-1,2,4-triazol-3-yl)methanone

$C_{23}H_{21}Cl_5N_4O_2$

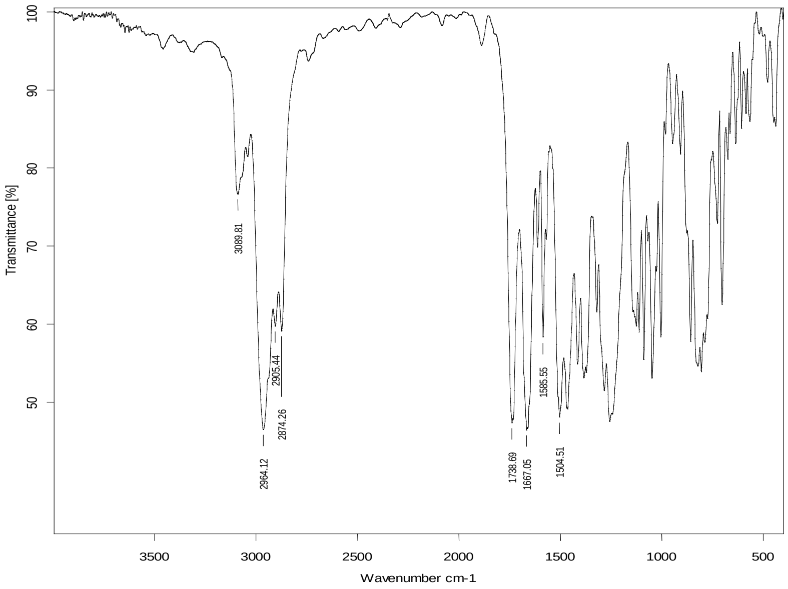

The IR spectrogram of compound **5h**

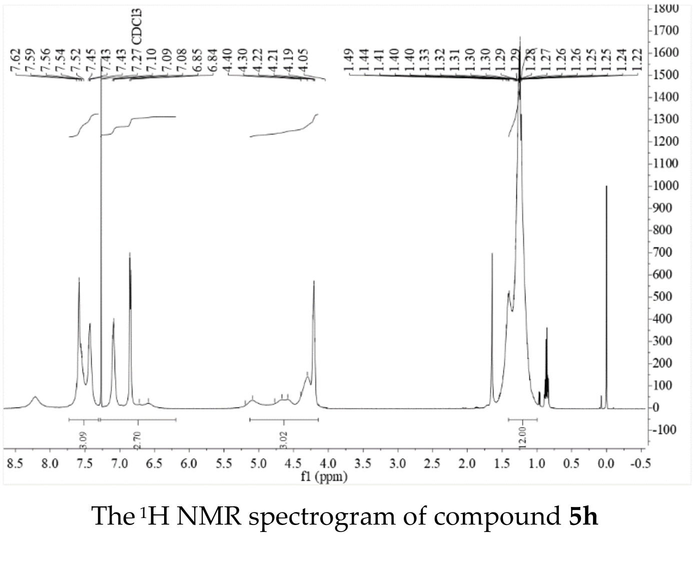

The ^1H NMR spectrogram of compound **5h**

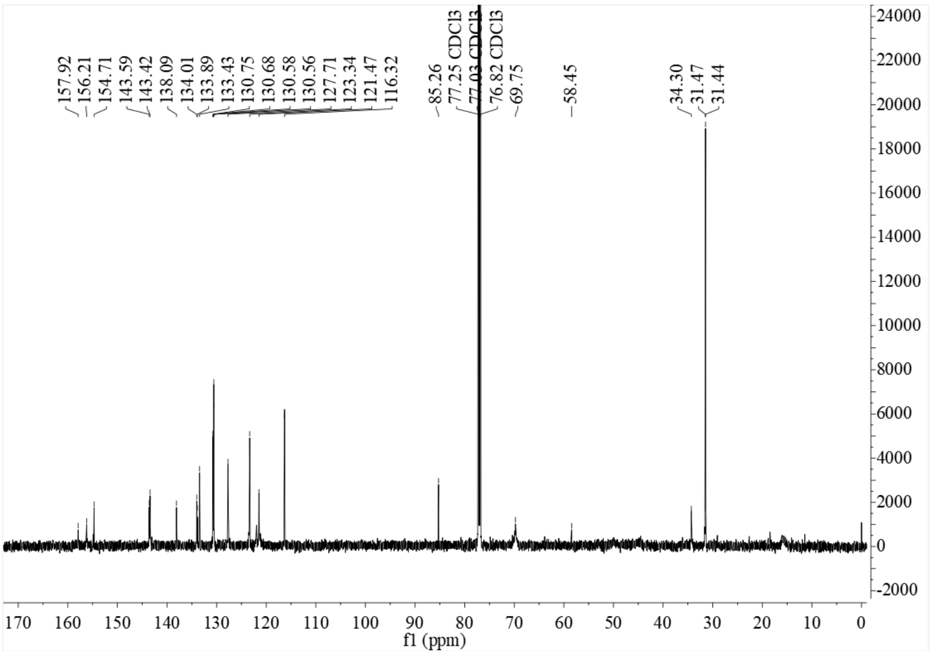

The ^{13}C NMR spectrogram of compound **5h**

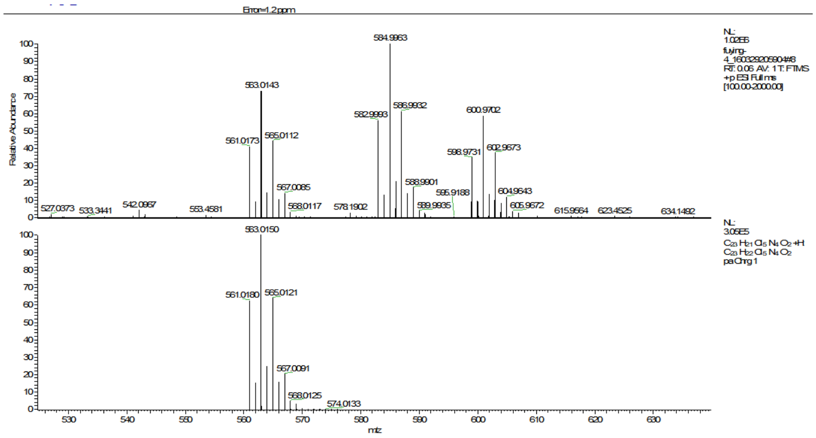

The HRMS spectrogram of compound **5h**

9. The molecular structure and spectra of representative compound **5i**

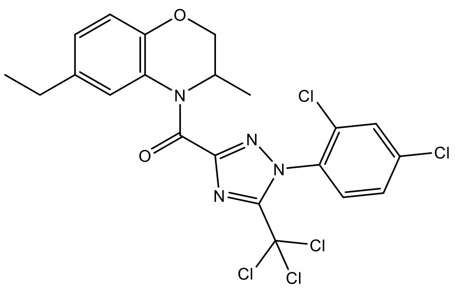

(1-(2,4-dichlorophenyl)-5-(trichloromethyl)-1*H*-1,2,4-triazol-3-yl)(6-ethyl-3-methyl-2,3-dihydro-4*H*-benzo[*b*][1,4]oxazin-4-yl)methanone

$C_{21}H_{17}Cl_5N_4O_2$

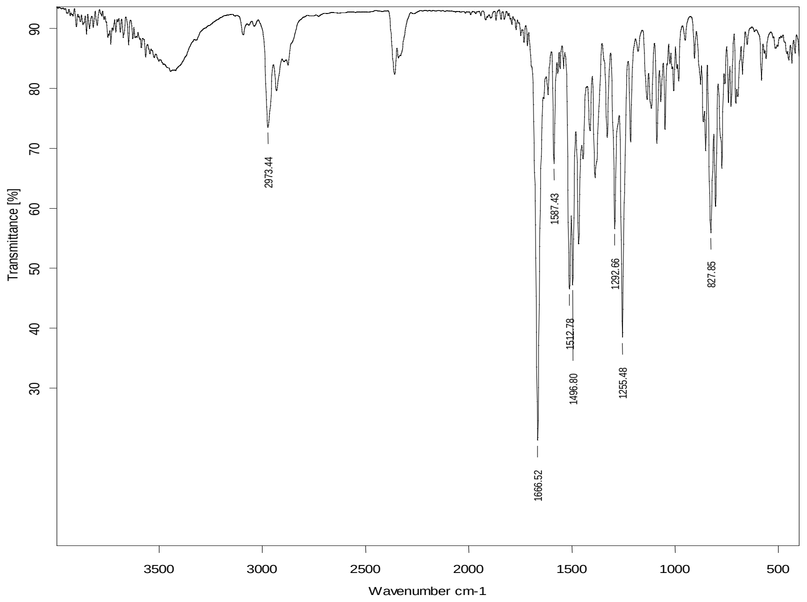

The IR spectrogram of compound **5i**

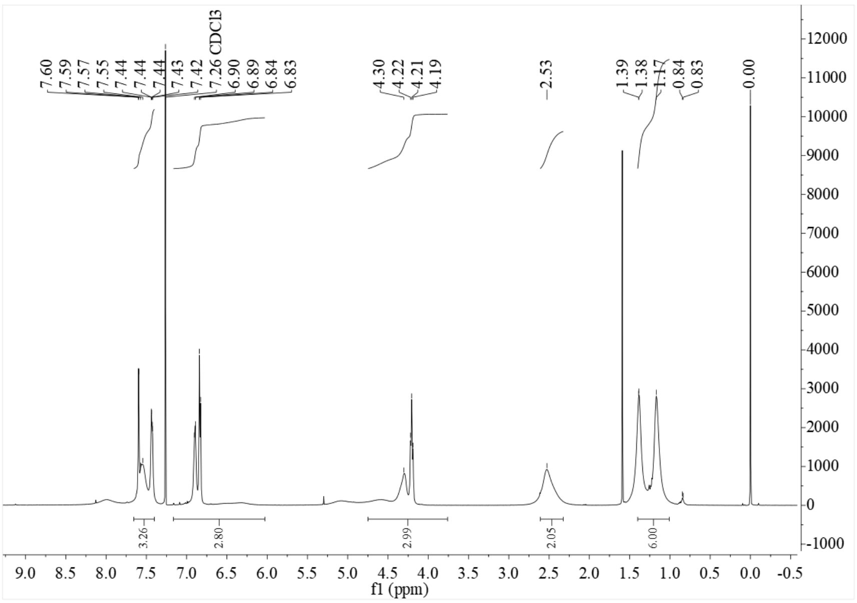

The ^{1}H NMR spectrogram of compound **5i**

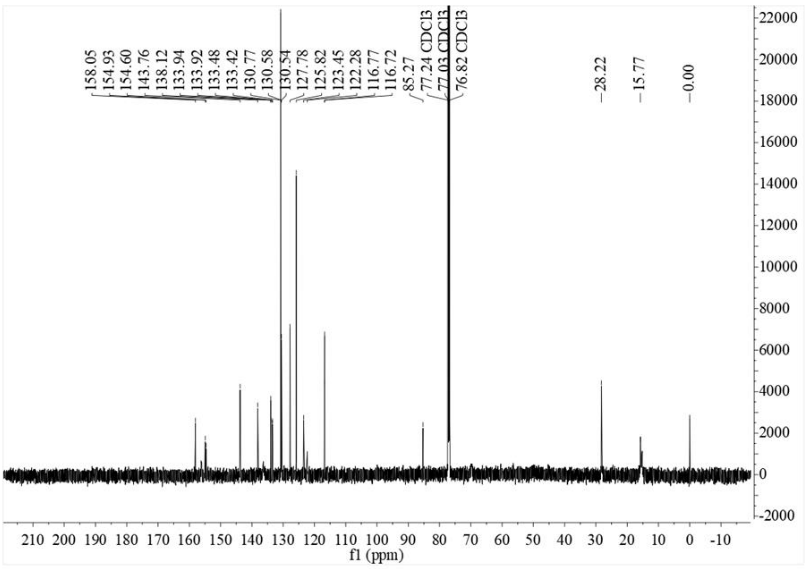

The ^{13}C NMR spectrogram of compound **5i**

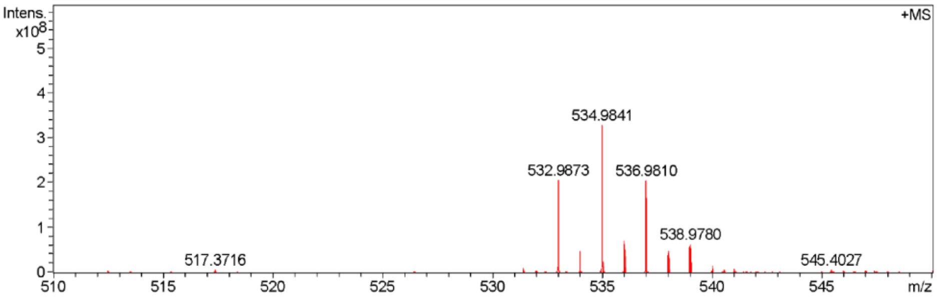

The HRMS spectrogram of compound **5i**

10. The molecular structure and spectra of representative compound **5j**

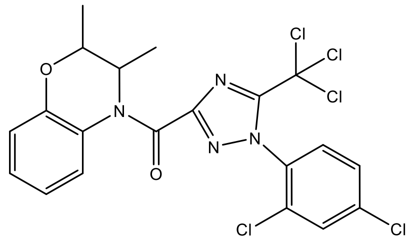

(1-(2,4-dichlorophenyl)-5-(trichloromethyl)-1*H*-1,2,4-triazol-3-yl)(2,3-dimethyl-2,3-dihydro-4*H*-benzo[*b*][1,4]oxazin-4-yl)methanone

$C_{20}H_{15}Cl_5N_4O_2$

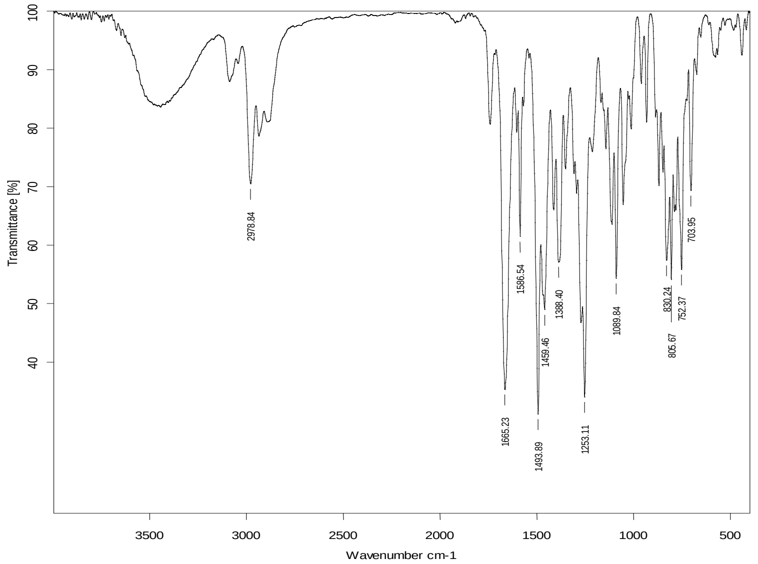

The IR spectrogram of compound **5j**

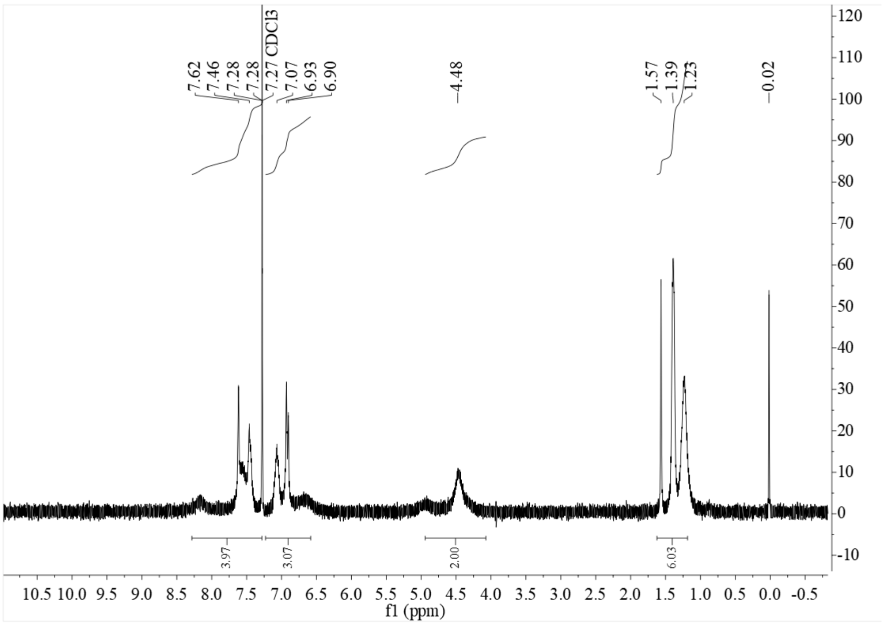

The ^{1}H NMR spectrogram of compound **5j**

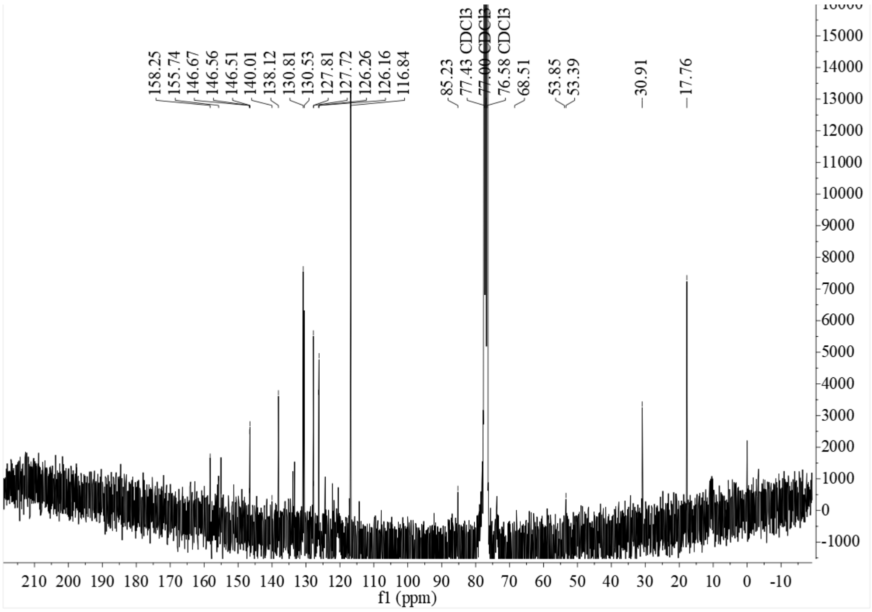

The ^{13}C NMR spectrogram of compound **5j**

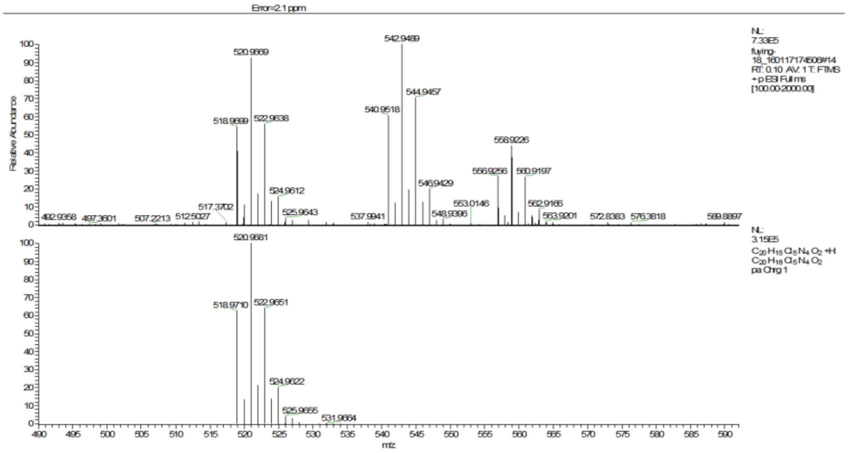

The HRMS spectrogram of compound **5j**

11. The molecular structure and spectra of representative compound **5k**

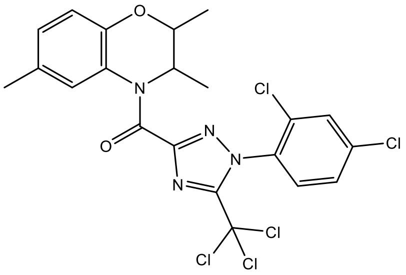

(1-(2,4-dichlorophenyl)-5-(trichloromethyl)-1*H*-1,2,4-triazol-3-yl)(2,3,6-trimethyl-2,3-dihydro-4*H*-benzo[*b*][1,4]oxazin-4-yl)methanone

$C_{21}H_{17}Cl_5N_4O_2$

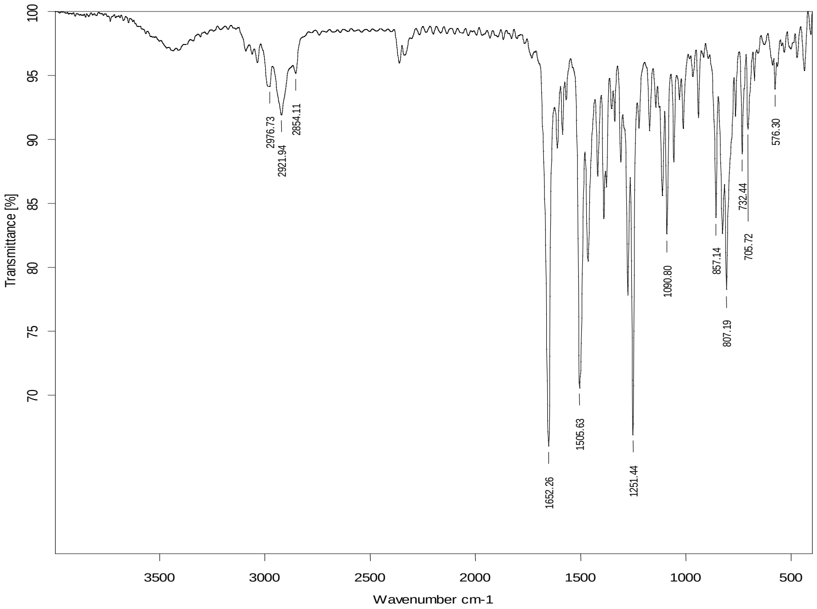

The IR spectrogram of compound **5k**

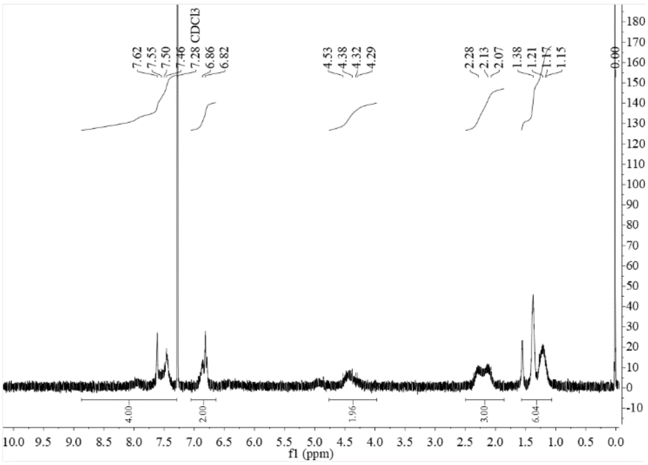

The ^{1}H NMR spectrogram of compound **5k**

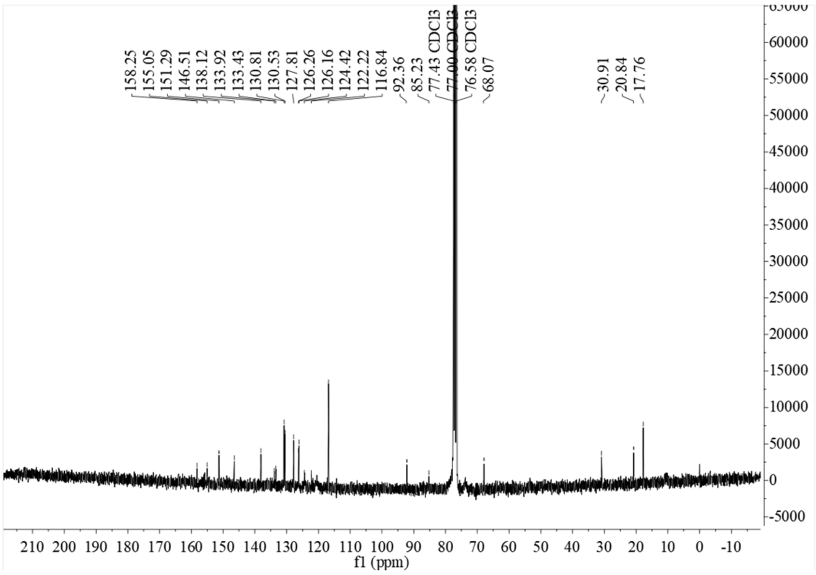

The ^{13}C NMR spectrogram of compound **5k**

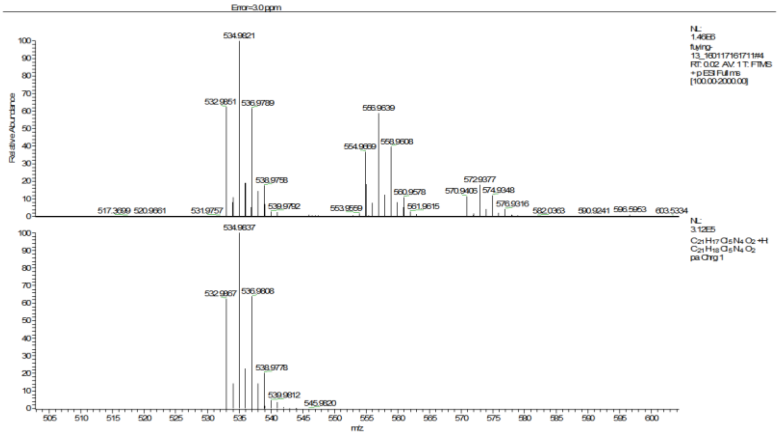

The HRMS spectrogram of compound **5k**

12. The molecular structure and spectra of representative compound **5l**

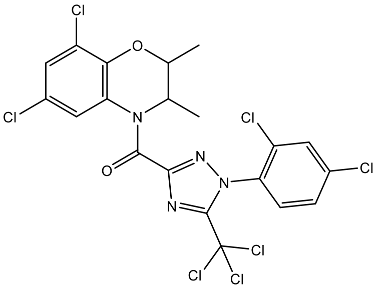

(6,8-dichloro-2,3-dimethyl-2,3-dihydro-4*H*-benzo[*b*][1,4]oxazin-4-yl)(1-(2,4-dichlorophenyl)-5-(trichloromethyl)-1*H*-1,2,4-triazol-3-yl)methanone

$C_{20}H_{13}Cl_7N_4O_2$

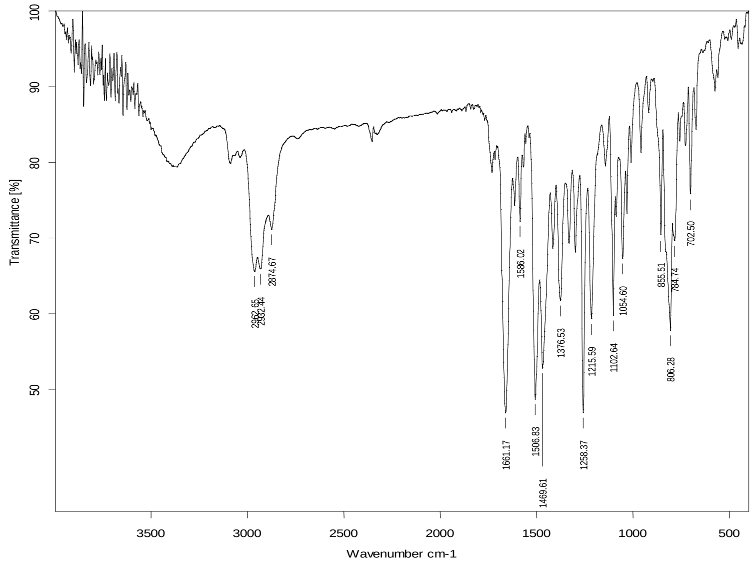

The IR spectrogram of compound **5l**

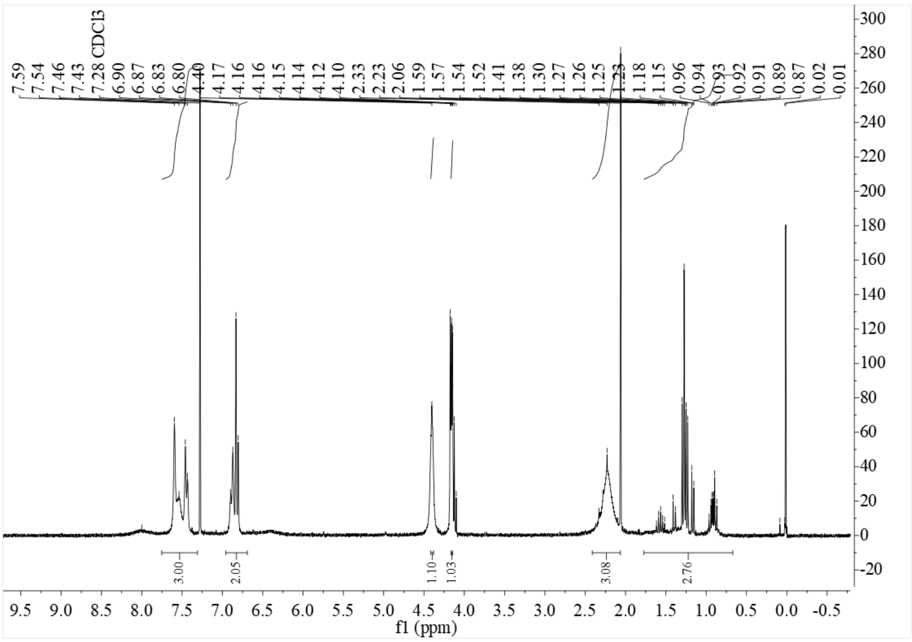

The ^{1}H NMR spectrogram of compound **5l**

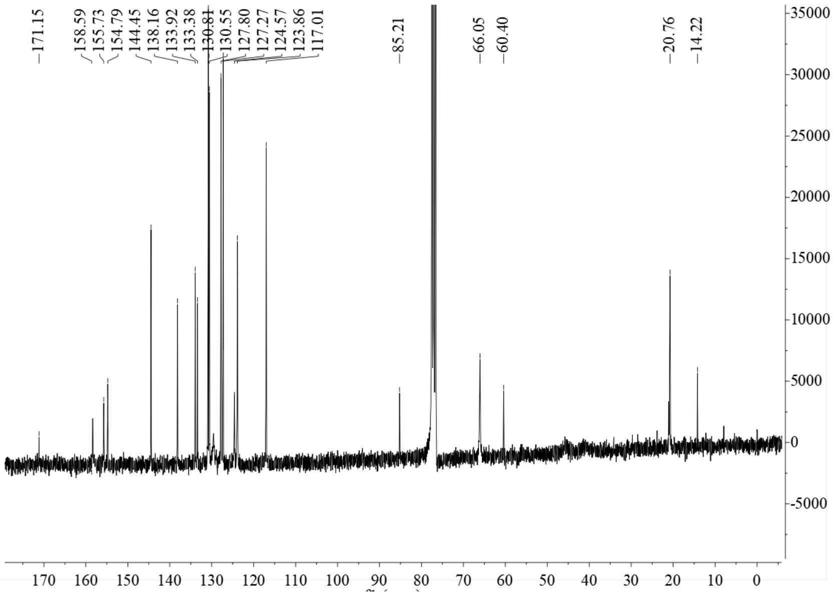

The ^{13}C NMR spectrogram of compound **5l**

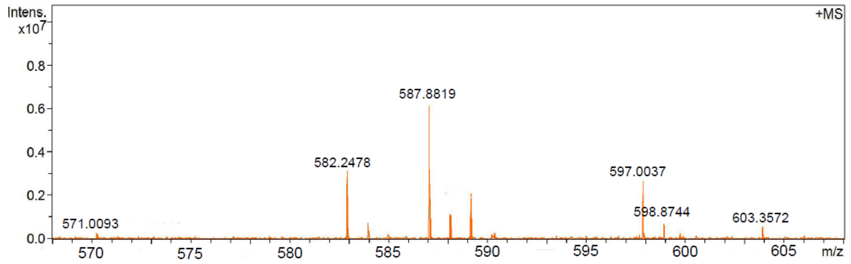

Meas. m/z	#	Formula	Score	m/z	err [ppm]	e¡¥Conf	N-Rule
587.8819	1	C 20 H 13 Cl 7 N 4 O 2	100.00	587.8820	-1.2	even	ok

The HRMS spectrogram of compound **5l**

13. The molecular structure and spectra of representative compound **5m**

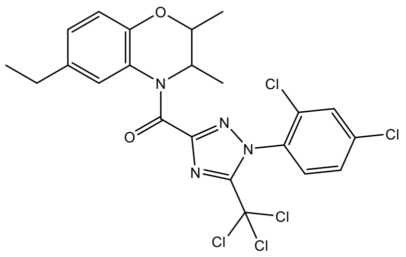

(1-(2,4-dichlorophenyl)-5-(trichloromethyl)-1*H*-1,2,4-triazol-3-yl)(6-ethyl-2,3-dimethyl-2,3-dihydro-4*H*-benzo[*b*][1,4]oxazin-4-yl)methanone

$C_{22}H_{19}Cl_5N_4O_2$

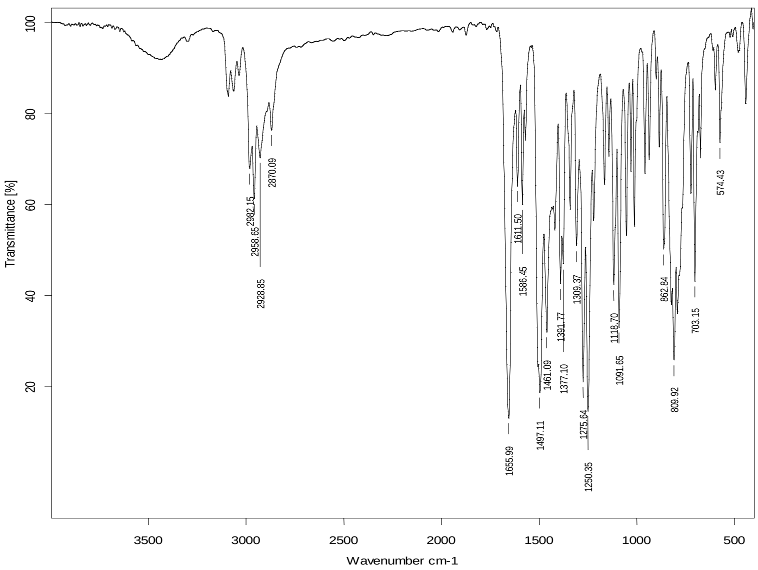

The IR spectrogram of compound **5m**

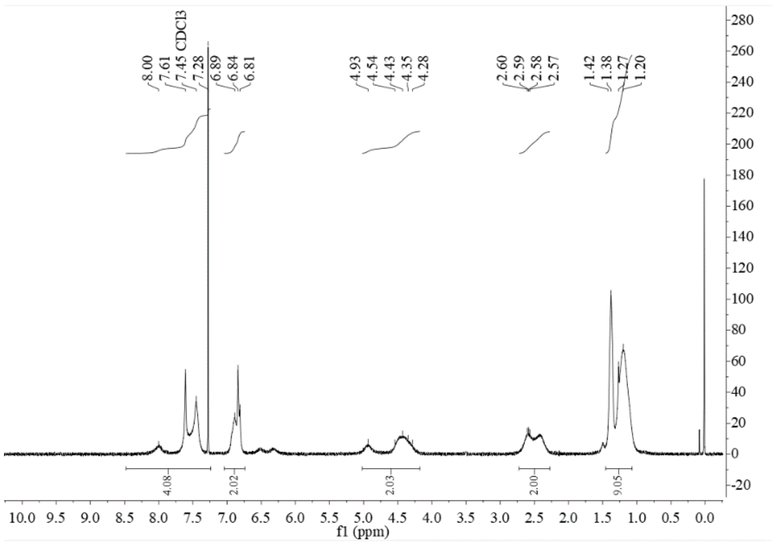

The ^{1}H NMR spectrogram of compound **5m**

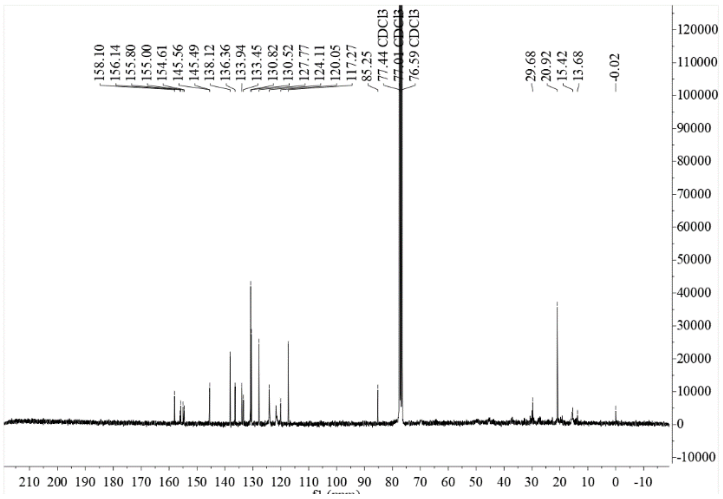

The ^{13}C NMR spectrogram of compound **5m**

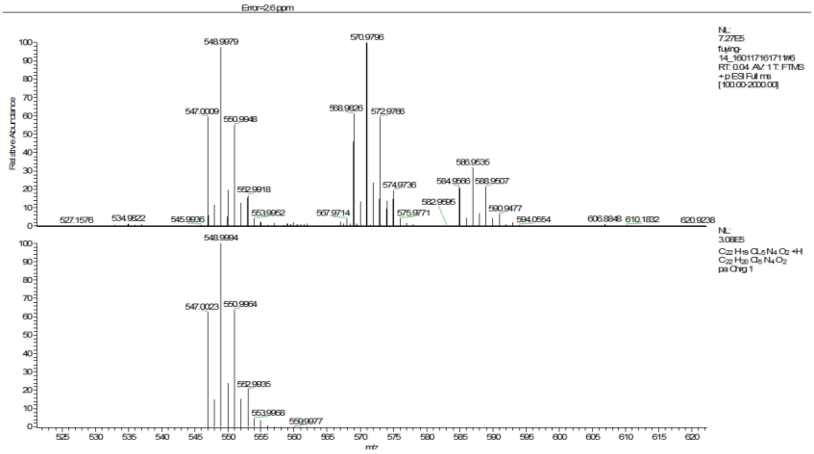

The HRMS spectrogram of compound **5m**

14. The molecular structure and spectra of representative compound **5n**

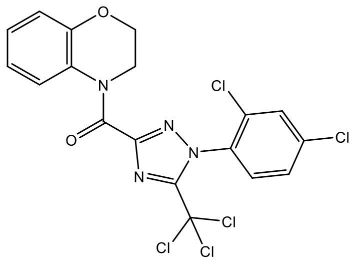

(1-(2,4-dichlorophenyl)-5-(trichloromethyl)-1*H*-1,2,4-triazol-3-yl)(2,3-dihydro-4*H*-benzo[*b*][1,4]oxazin-4-yl)methanone

$C_{18}H_{11}Cl_5N_4O_2$

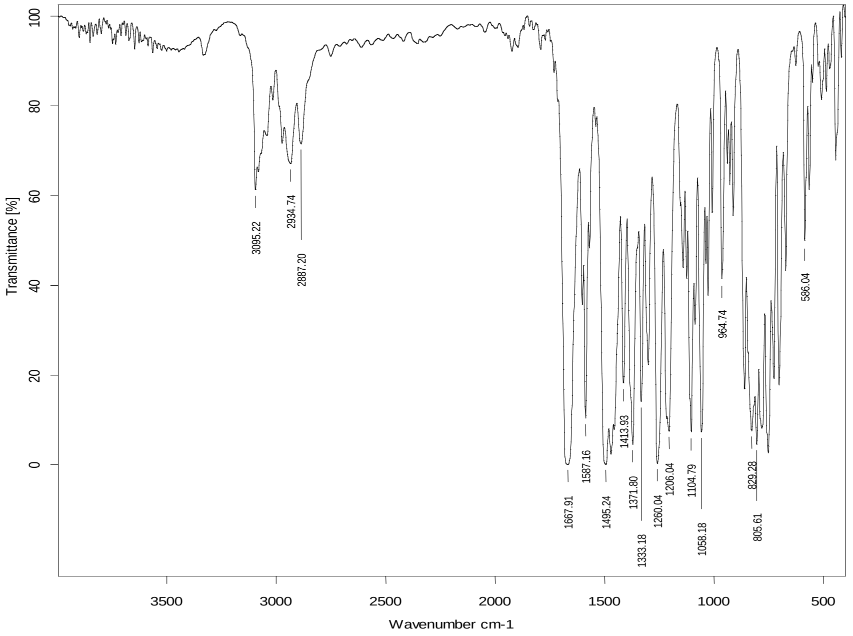

The IR spectrogram of compound **5n**

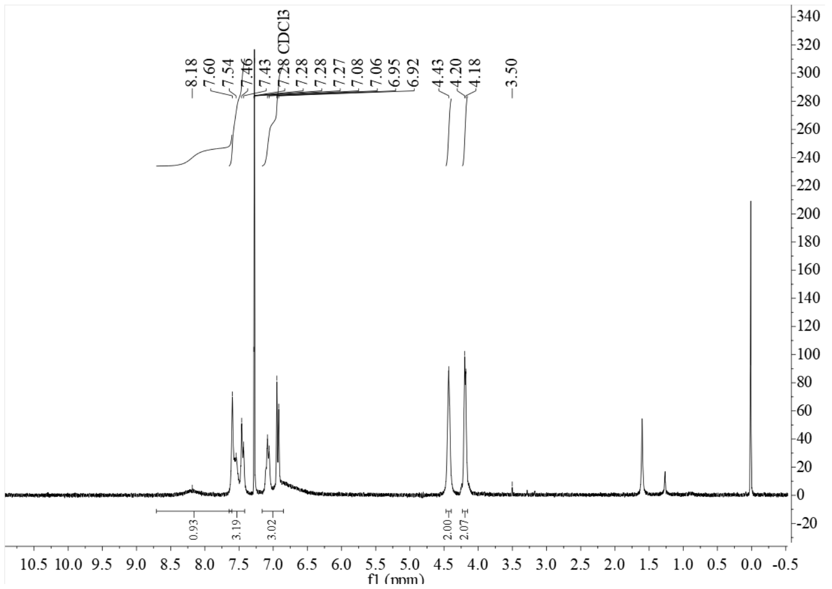

The ^{1}H NMR spectrogram of compound **5n**

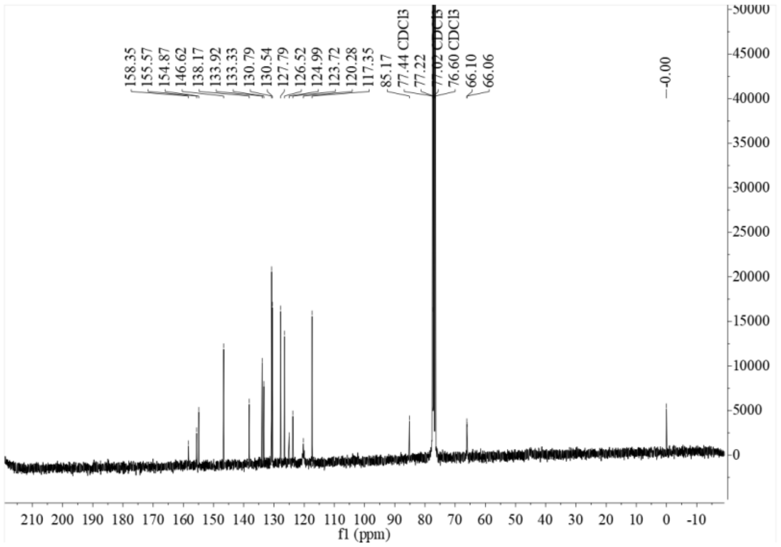

The ^{13}C NMR spectrogram of compound **5n**

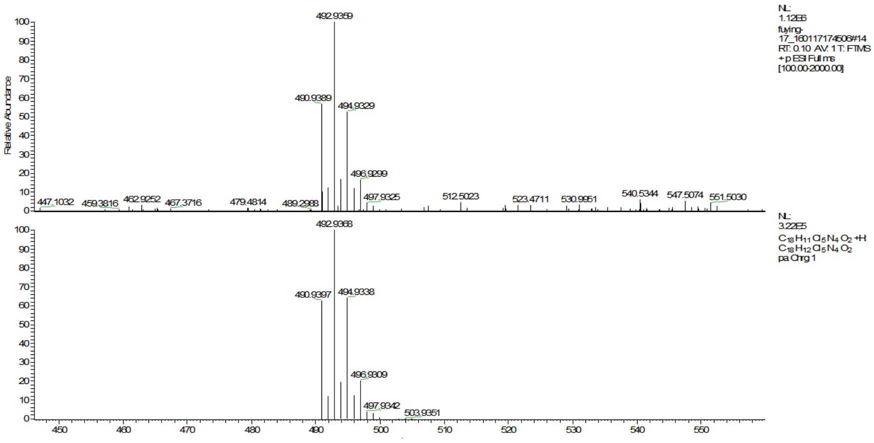

The HRMS spectrogram of compound **5n**

15. The molecular structure and spectra of representative compound **5o**

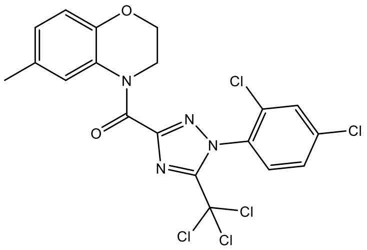

(1-(2,4-dichlorophenyl)-5-(trichloromethyl)-1*H*-1,2,4-triazol-3-yl)(6-methyl-2,3-dihydro-4*H*-benzo[*b*][1,4]oxazin-4-yl)methanone

$C_{19}H_{13}Cl_5N_4O_2$

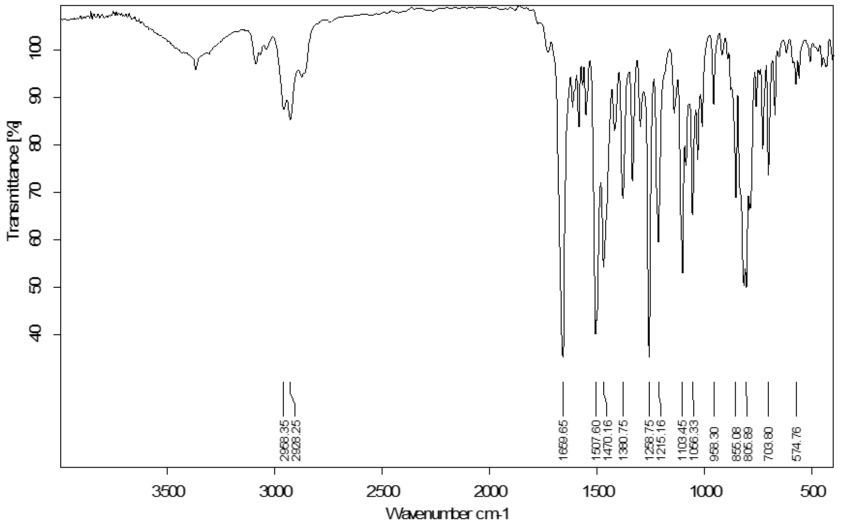

The IR spectrogram of compound **5o**

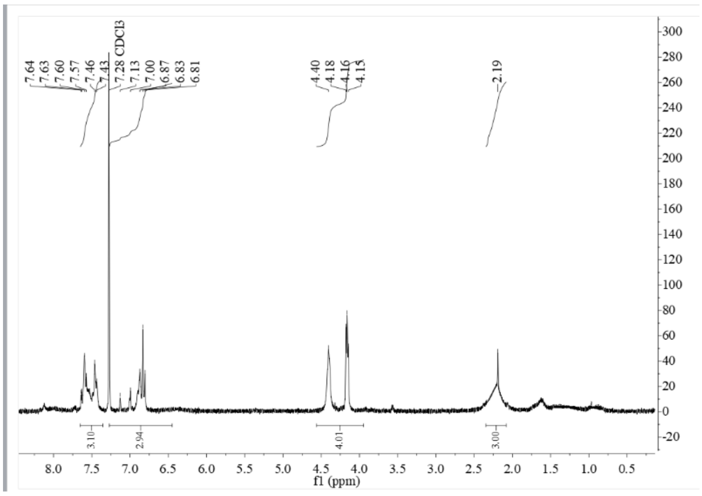

The ^{1}H NMR spectrogram of compound **5o**

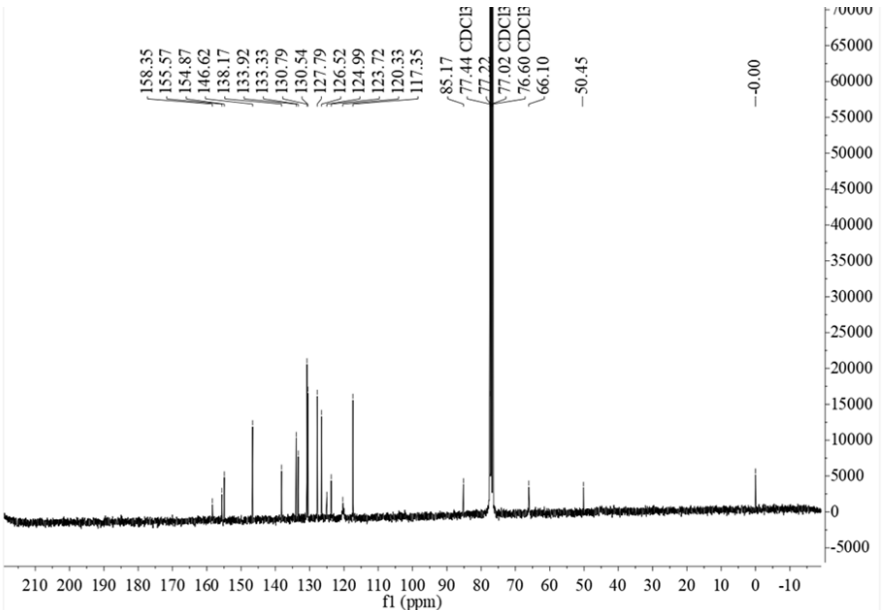

The ^{13}C NMR spectrogram of compound **5o**

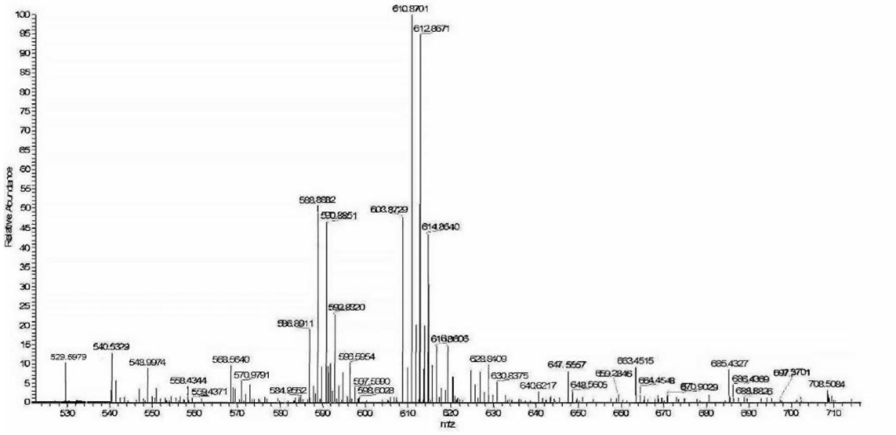

The HRMS spectrogram of compound **5o**

16. The molecular structure and spectra of representative compound **5p**

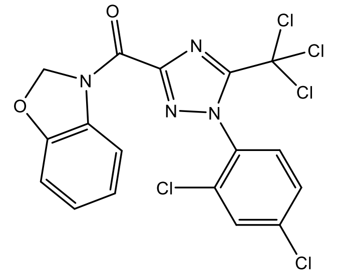

benzo[*d*]oxazol-3(2*H*)-yl(1-(2,4-dichlorophenyl)-5-(trichloromethyl)-1*H*-1,2,4-triazol-3-yl)methanone

$C_{17}H_9Cl_5N_4O_2$

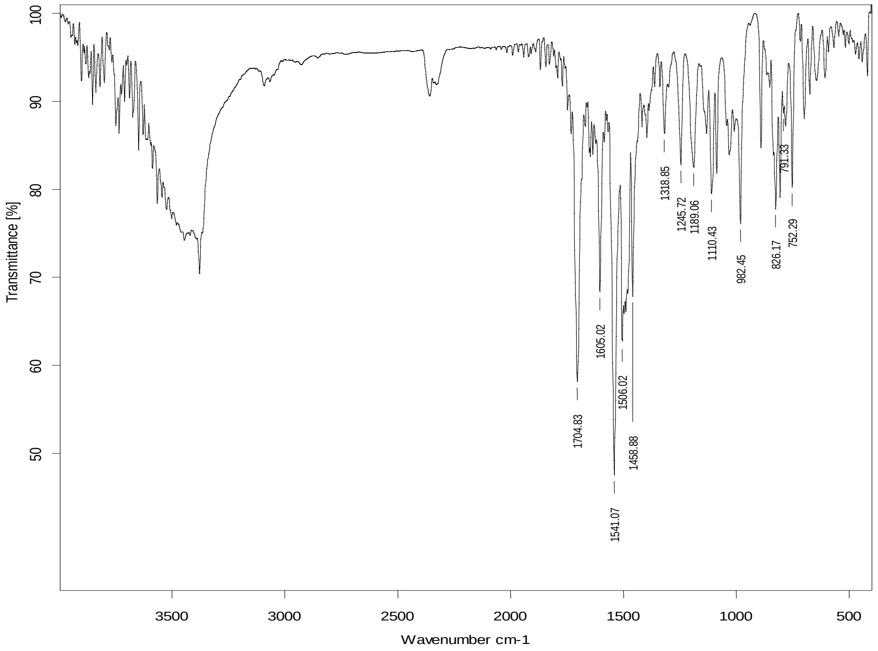

The IR spectrogram of compound **5p**

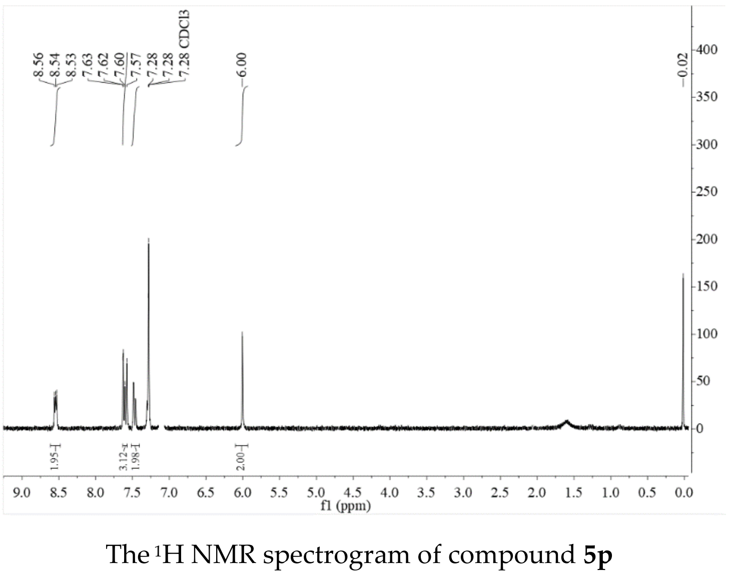

The ^{1}H NMR spectrogram of compound **5p**

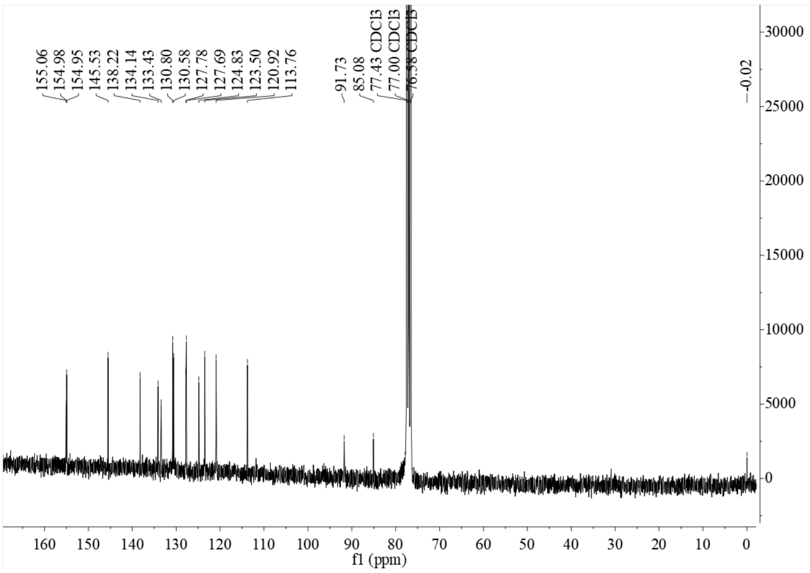

The ^{13}C NMR spectrogram of compound **5p**

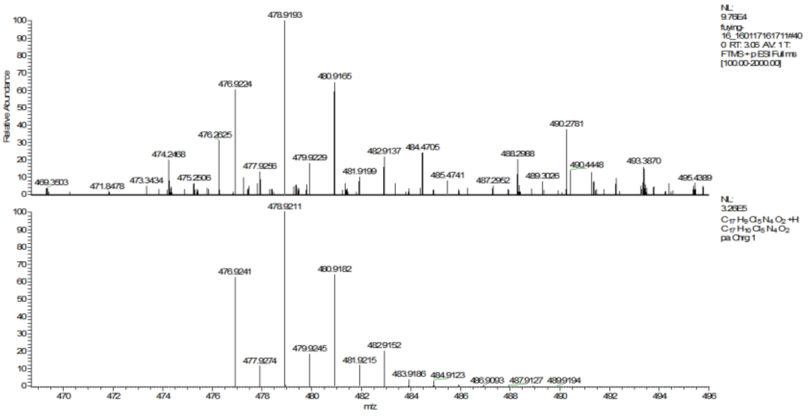

The HRMS spectrogram of compound **5p**

17. The molecular structure and spectra of representative compound **5q**

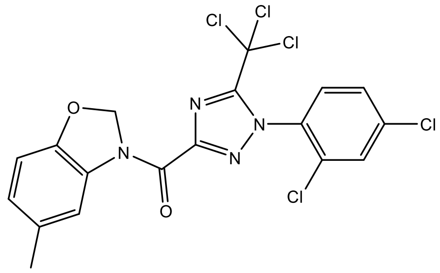

(1-(2,4-dichlorophenyl)-5-(trichloromethyl)-1*H*-1,2,4-triazol-3-yl)(5-methylbenzo[*d*]oxazol-3(2*H*)-yl)methanone

$C_{18}H_{11}Cl_5N_4O_2$

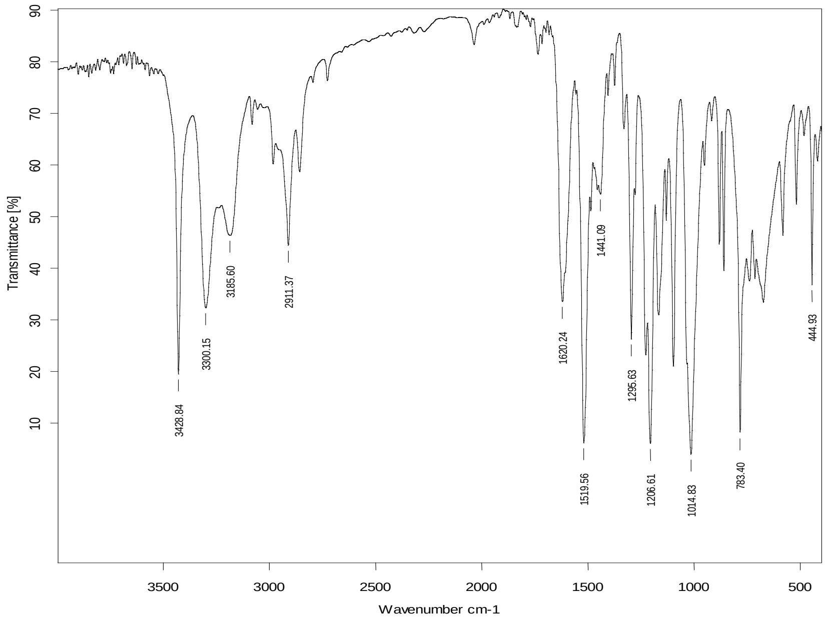

The IR spectrogram of compound **5q**

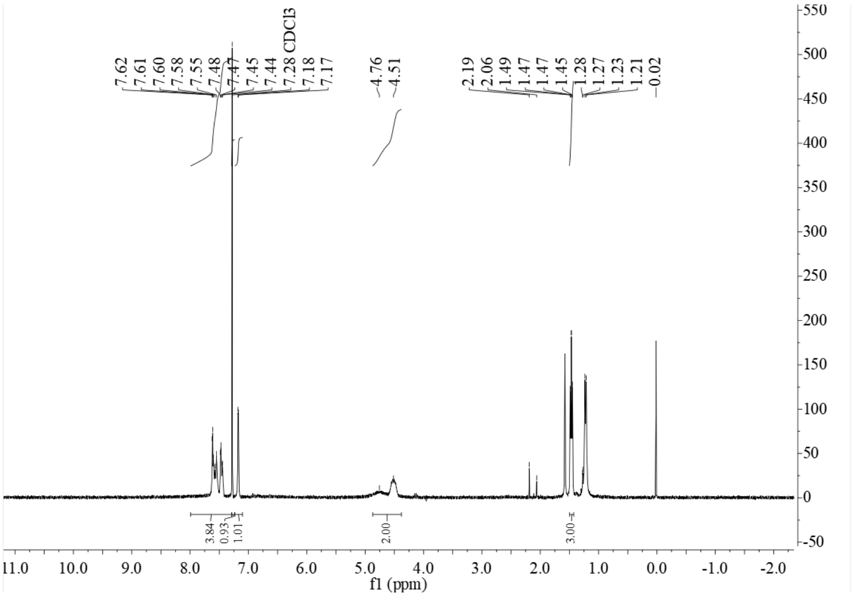

The ^{1}H NMR spectrogram of compound **5q**

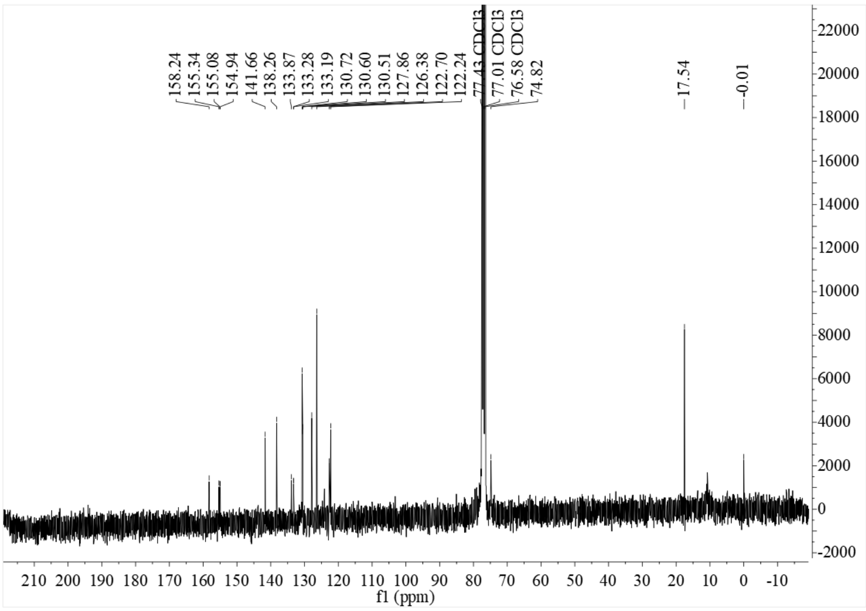

The ^{13}C NMR spectrogram of compound **5q**

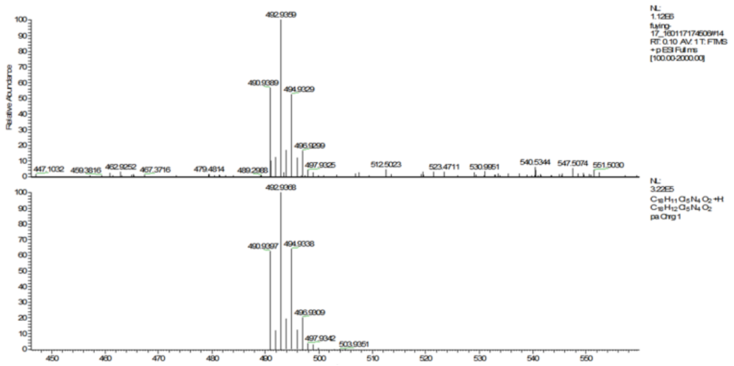

The HRMS spectrogram of compound **5q**

18. The molecular structure and spectra of representative compound **5r**

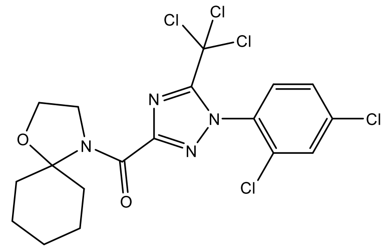

(1-(2,4-dichlorophenyl)-5-(trichloromethyl)-1*H*-1,2,4-triazol-3-yl)(1-oxa-4-azaspiro[4.5]decan-4-yl)methanone

$C_{18}H_{17}Cl_5N_4O_2$

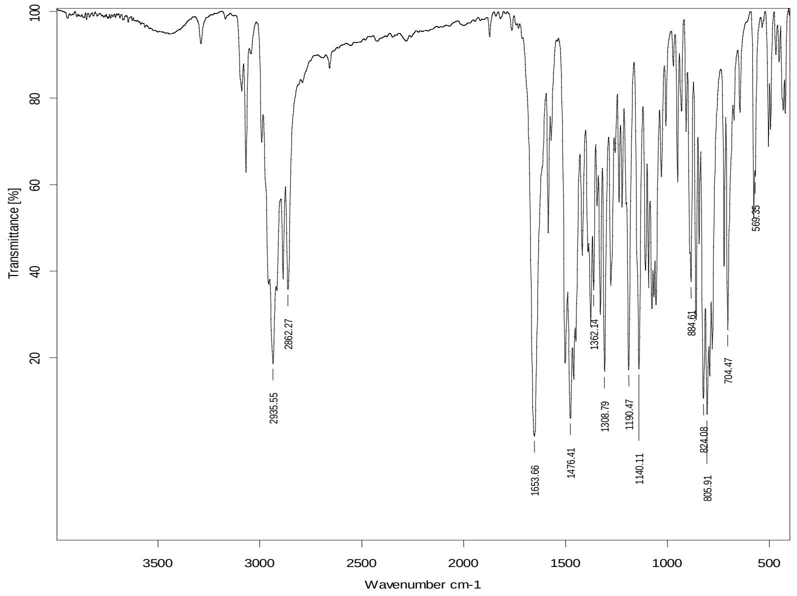

The IR spectrogram of compound **5r**

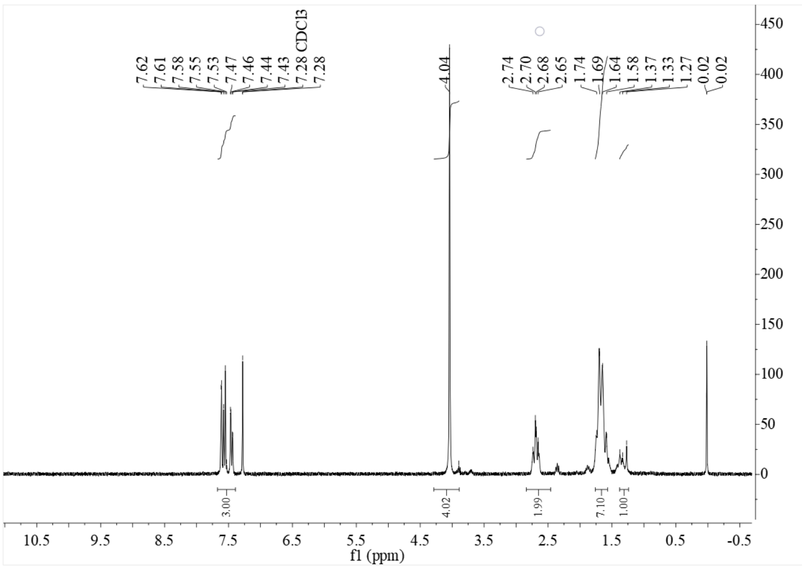

The ^{1}H NMR spectrogram of compound **5r**

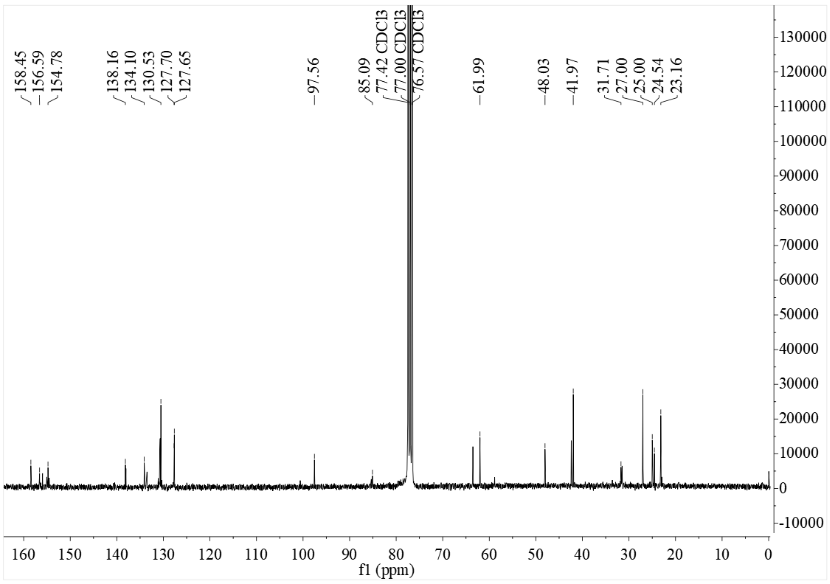

The ^{13}C NMR spectrogram of compound **5r**

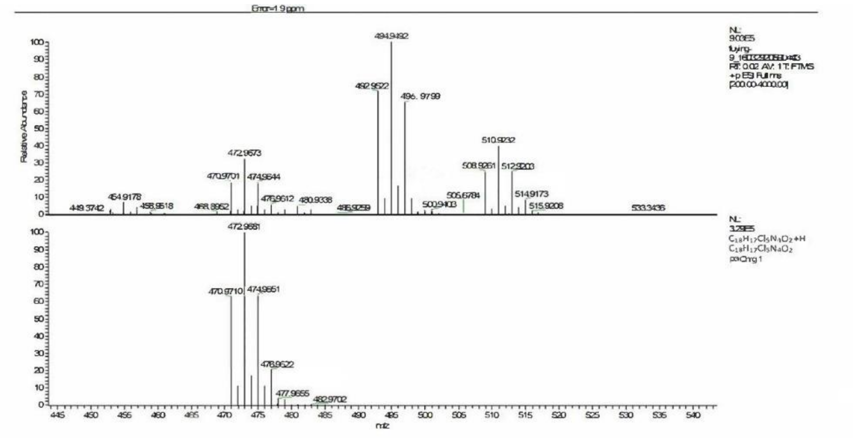

The HRMS spectrogram of compound **5r**

19. The molecular structure and spectra of representative compound **5s**

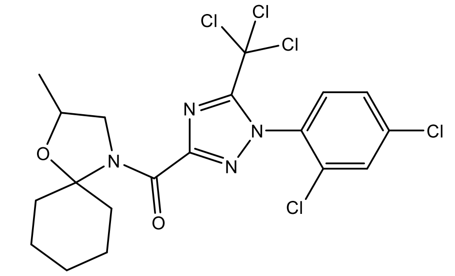

(1-(2,4-dichlorophenyl)-5-(trichloromethyl)-1*H*-1,2,4-triazol-3-yl)(2-methyl-1-oxa-4-azaspiro[4.5]decan-4-yl)methanone

$C_{19}H_{19}Cl_5N_4O_2$

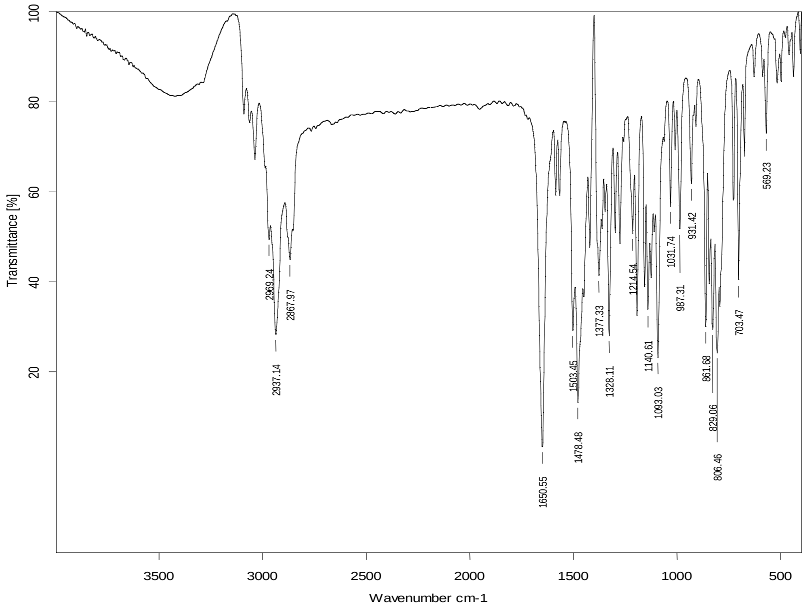

The IR spectrogram of compound **5s**

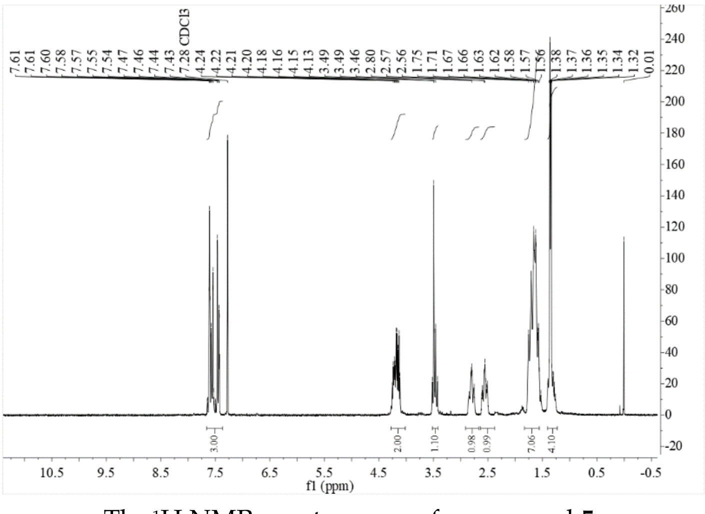

The ^{1}H NMR spectrogram of compound **5s**

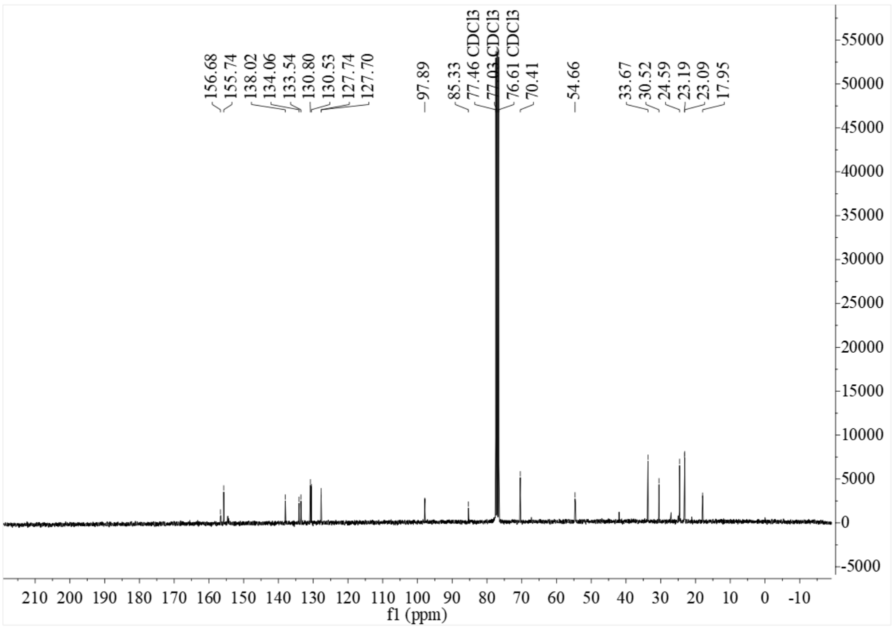

The ^{13}C NMR spectrogram of compound **5s**

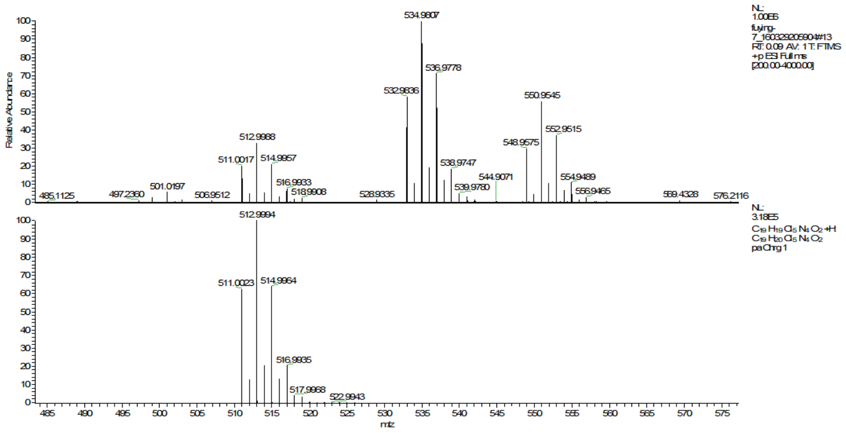

The HRMS spectrogram of compound **5s**

20. The molecular structure and spectra of representative compound **5t**

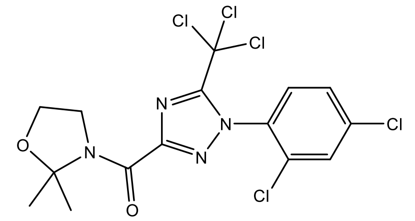

(1-(2,4-dichlorophenyl)-5-(trichloromethyl)-1*H*-1,2,4-triazol-3-yl)(2,2-dimethyloxazolidin-3-yl)methanone

$C_{15}H_{13}Cl_5N_4O_2$

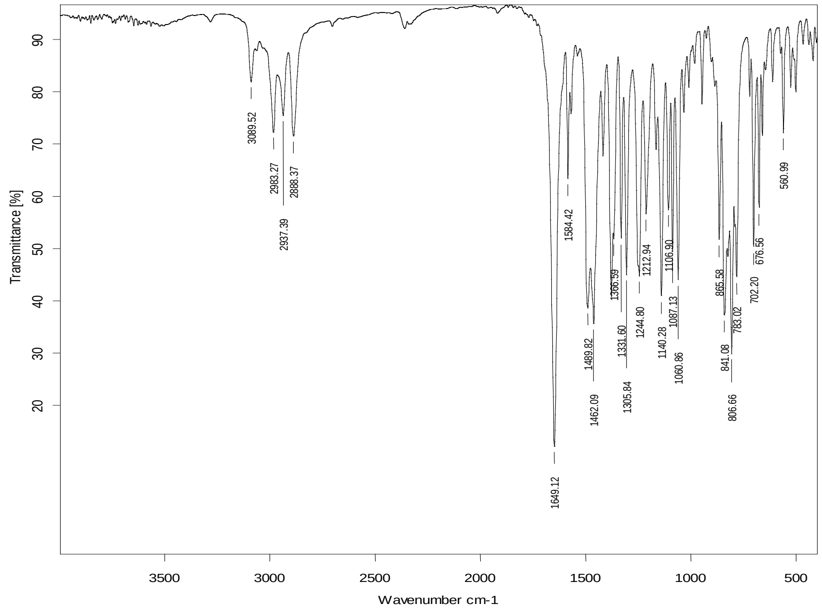

The IR spectrogram of compound **5t**

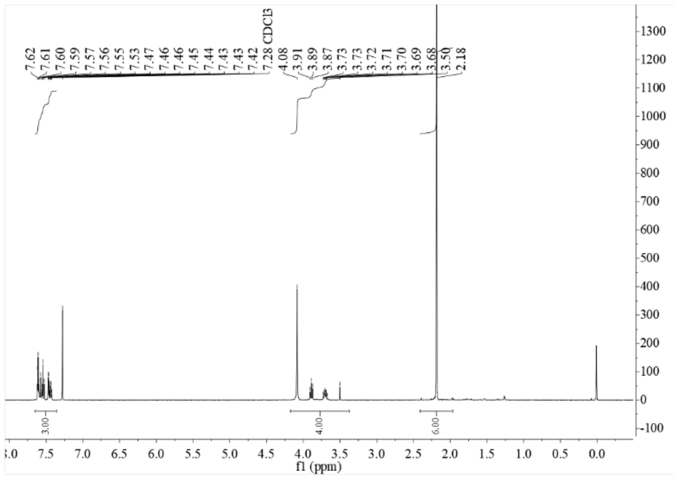

The ^{1}H NMR spectrogram of compound **5t**

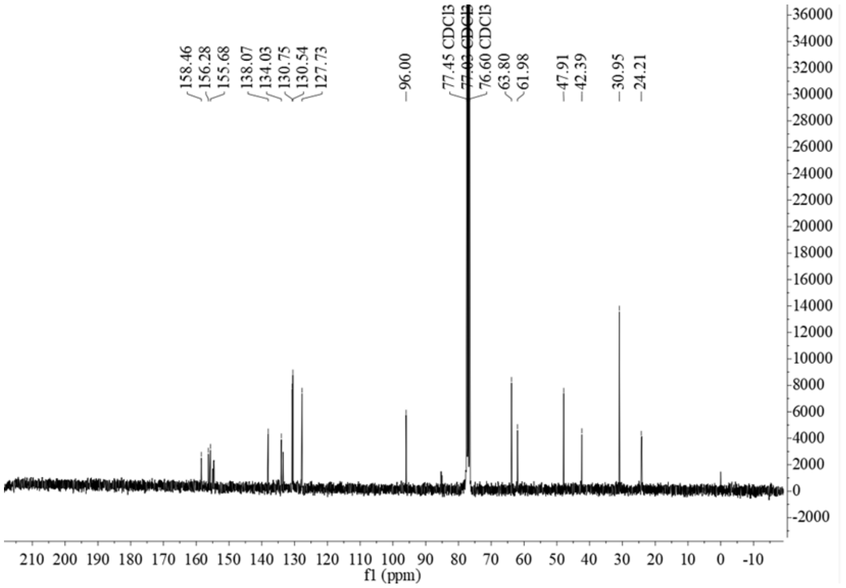

The ^{13}C NMR spectrogram of compound **5t**

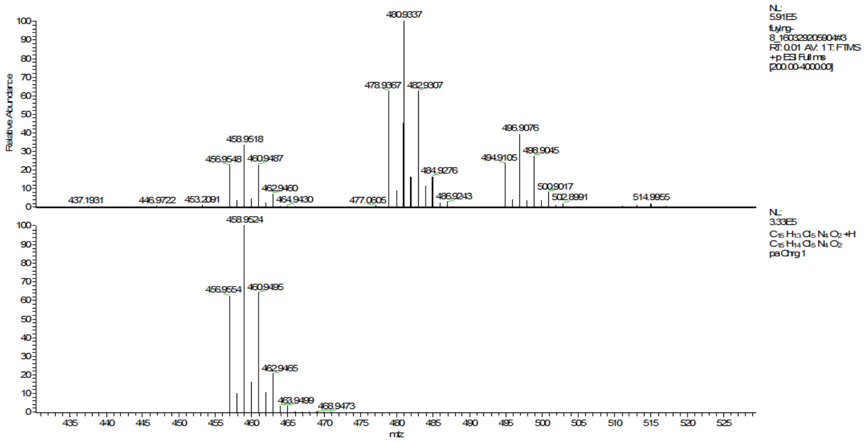

The HRMS spectrogram of compound **5t**

21. The molecular structure and spectra of representative compound **5u**

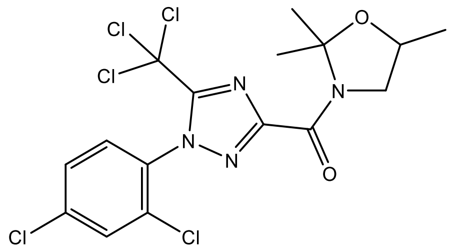

(1-(2,4-dichlorophenyl)-5-(trichloromethyl)-1*H*-1,2,4-triazol-3-yl)(2,2,5-trimethyloxazolidin-3-yl)methanone

$C_{16}H_{15}Cl_5N_4O_2$

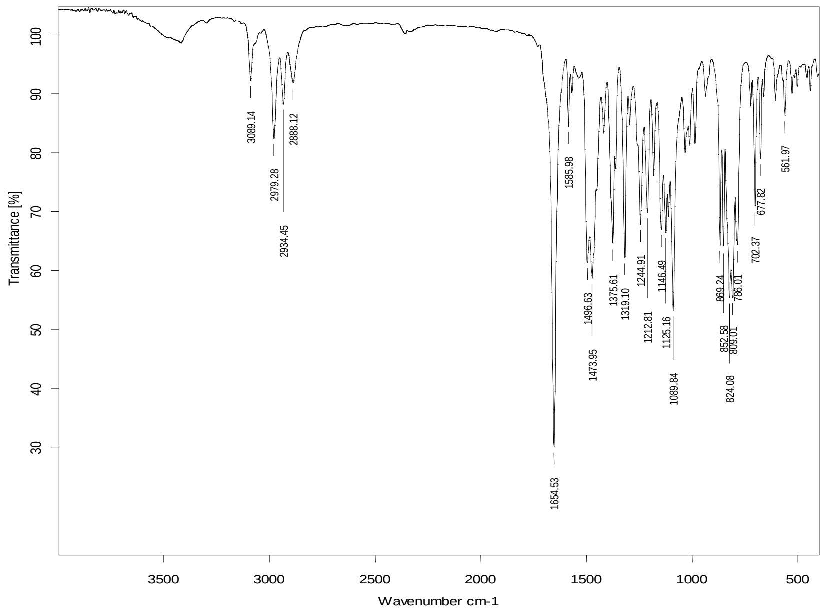

The IR spectrogram of compound **5u**

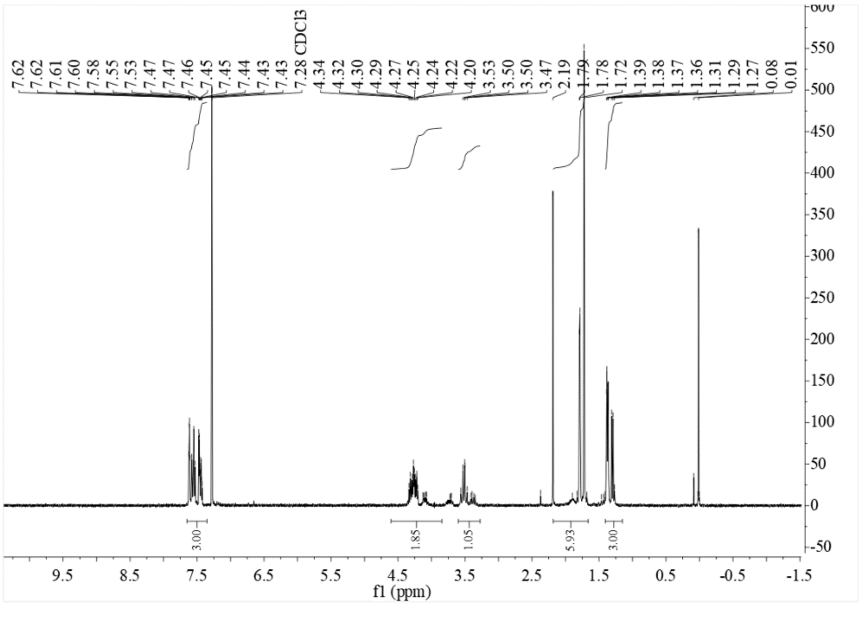

The ^{1}H NMR spectrogram of compound **5u**

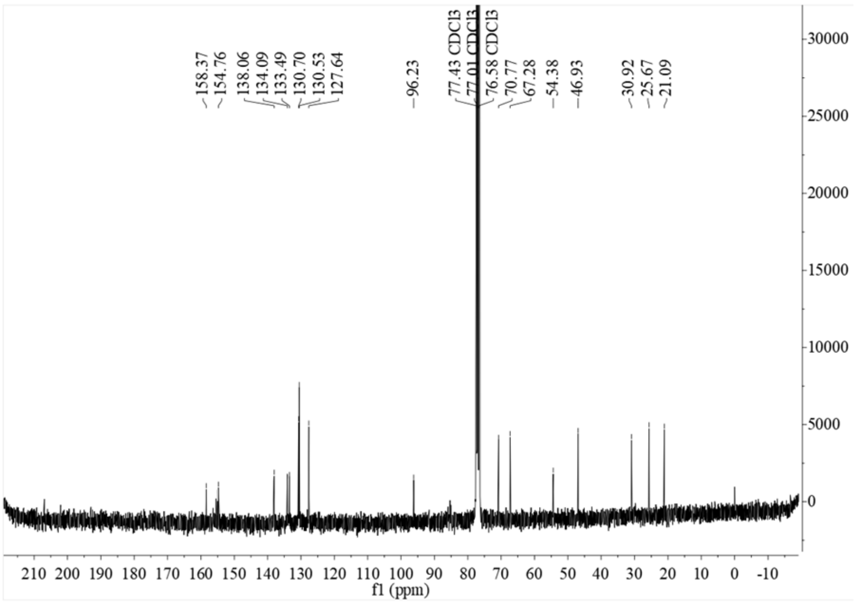

The ^{13}C NMR spectrogram of compound **5u**

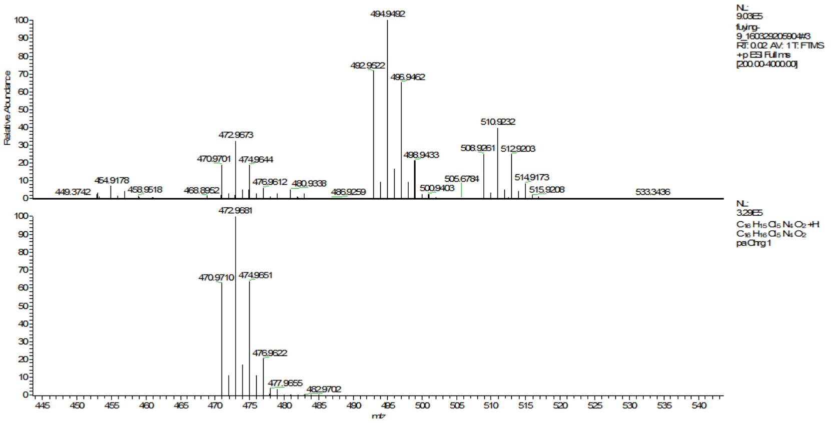

The HRMS spectrogram of compound **5u**
